# CHIR99021 Treatment Promotes Pigmentation but Differentially Affects RPE‐Associated Transcriptional Programs in Human iPSC‐Derived RPE

**DOI:** 10.1002/cbf.70343

**Published:** 2026-11-15

**Authors:** Gopika S. Kumar, Srijan Jayaraman, Anwar A. Palakkan

**Affiliations:** ^1^ Department of Immunology and Stem Cell Biology Aravind Medical Research Foundation, Anna Nagar Madurai India; ^2^ Abate Eye Hospital Building Perinthalmanna Malappuram Kerala India

**Keywords:** CHIR99021, iPSC‐derived RPE, quantification of pigmentation, retinal pigment epithelium, RPE maturation, RPE pigmentation

## Abstract

Maintenance of epithelial identity and RPE‐associated characteristics is important for the long‐term utility of induced pluripotent stem cell‐derived retinal pigment epithelium (iRPE) cells in disease modelling and regenerative applications. However, the effects of prolonged modulation of developmental signalling pathways on the phenotype and transcriptional state of differentiated iRPE cells remain incompletely understood. In this study, we investigated the effects of small‐molecule modulation of developmental signalling pathways on iRPE differentiation and long‐term culture. Small‐molecule screening identified CHIR99021 as the most effective treatment condition among those tested for promoting the generation of pigment‐positive iRPE cells. CHIR99021 treatment enhanced relative pigmentation and was associated with the retention of pigmentation and epithelial‐associated characteristics during extended culture and serial passaging. Transcriptomic analyses revealed changes in programmes associated with pigmentation, epithelial organisation, lysosomal activity, lipid metabolism, and cell‐cycle regulation. In contrast, transcriptional programmes associated with several specialised RPE functions, including visual‐cycle activity, phagocytosis, microvilli, and ciliogenesis, were not uniformly enhanced and in several cases showed reduced expression following CHIR99021 treatment. Analysis of a foetal retinal pigment cell‐associated gene set further supported an overall tendency toward reduced expression of RPE‐associated genes following CHIR99021 treatment. These findings indicate that CHIR99021 treatment can enhance or preserve pigmentation and is associated with epithelial‐related characteristics without uniformly supporting broader transcriptional programmes associated with specialised RPE functions. Our findings highlight that pigmentation alone does not necessarily reflect broader RPE maturation and emphasise the importance of integrating morphological, molecular, and functional assessments when evaluating iRPE maturation and long‐term culture conditions.

## Significance

Maintenance of epithelial identity is essential for the long‐term stability and functionality of induced pluripotent stem cell‐derived retinal pigment epithelium (iRPE). This study demonstrates that CHIR99021 treatment promotes pigmentation and epithelial ‐associated characteristics during extended iRPE culture, while incompletely supporting specialized RPE functions, highlighting the distinction between pigmentary/epithelial phenotypes and functional RPE maturation. We also establish a simple image‐based method for semi‐quantitative assessment of relative optical pigmentation using a melanin reference. These findings provide insights into the differential regulation of pigmentary, epithelial, and specialized functional characteristics in iRPE cultures and provide practical approaches for evaluating iRPE phenotypes during prolonged culture.

## 1 Introduction

The retinal pigment epithelium (RPE) is a highly specialized, pigmented epithelial monolayer that plays a central role in maintaining retinal homeostasis and visual function. Strategically positioned between the photoreceptors and the choroidal vasculature, the RPE supports photoreceptor survival through a diverse array of essential functions, including absorption of excess light, phagocytosis of shed photoreceptor outer segments, recycling of visual cycle intermediates, maintenance of the outer blood–retinal barrier, and protection against oxidative stress and photo‐oxidative damage [1]. Given its constant exposure to high metabolic demand, light, and reactive oxygen species, the RPE is particularly vulnerable to age‐ and stress‐related dysfunction. Accordingly, degeneration or loss of RPE integrity represents a central pathological hallmark of several retinal disorders, most notably age‐related macular degeneration (AMD), a leading cause of irreversible blindness worldwide [2].

Human induced pluripotent stem cell (iPSC)‐derived RPE (iRPE) cells have emerged as a powerful and versatile platform for modelling retinal disease mechanisms, screening therapeutic compounds, and developing regenerative strategies. Despite significant advances in differentiation protocols, achieving robust, homogeneous differentiation together with long‐term functional maintenance of iRPE cells in vitro remains a major challenge [3]. Prolonged culture and repeated passaging often lead to loss of epithelial identity, reduced pigmentation, and the emergence of heterogeneous or partially differentiated cell populations, thereby limiting reproducibility and translational applicability.

Wnt/β‐catenin signalling is a highly conserved developmental pathway with well‐established roles in eye morphogenesis, RPE specification, and epithelial fate determination [4]. During early development, tightly regulated Wnt activity governs lineage commitment toward the RPE [5]. However, the contribution of Wnt signalling beyond initial specification—particularly in relation to RPE maturation, pigmentation stability, and long‐term maintenance of epithelial identity—remains incompletely understood. Pigmentation, driven by the accumulation of melanin within melanosomes, is a defining hallmark of mature RPE and is frequently used as a surrogate indicator of differentiation status. Nevertheless, standardized quantitative approaches for assessing pigmentation are lacking, and the relationship between pigmentation and functional RPE maturity remains poorly defined.

In this study, we investigated the effects of CHIR99021 treatment during iPSC differentiation toward the RPE lineage and examined the effects of CHIR99021 treatment during long‐term maintenance and serial passaging of iRPE cultures. Using a standardized, non‐ destructive approach for semi‐quantitative assessment of relative pigmentation together with transcriptomic analyses, we evaluated how prolonged CHIR99021 treatment influences pigmentation, epithelial‐associated characteristics, and transcriptional programmes during extended culture. We further examined transcriptional programmes associated with specialised RPE functions and analysed the expression of a foetal retinal pigment cell‐associated gene set to determine whether enhanced pigmentation was accompanied by broader RPE‐associated transcriptional changes. Together, our findings show that CHIR99021 treatment promotes or preserves pigmentation and is associated with epithelial‐related characteristics, while not uniformly supporting transcriptional programmes associated with specialised RPE functions. These findings indicate that increased pigmentation does not necessarily reflect broader RPE maturation.

## 2 Materials and Methods

### 2.1 Human iPSC Culture

iPSC lines were used in this study because they provide a physiologically relevant model of RPE compared with immortalized RPE cell lines. The D2C and D3C iPSC lines were obtained as research donations from Dr. Kapil Bharti, National Eye Institute, National Institutes of Health, USA, in 2024. The D149 iPSC line was obtained from the Accelerator Program for Discovery in Brain Disorders using Stem Cells (ADBS) Biorepository, Institute for Stem Cell Biology and Regenerative Medicine, Bengaluru, India, in 2021. Research Resource Identifiers (RRIDs) are not currently available for these cell lines. Cell line identity was verified by the source laboratories using short tandem repeat (STR) profiling and showed greater than 90% concordance with the corresponding reference profiles. To the best of our knowledge, these cell lines have not been reported as misidentified or contaminated. All experiments were performed using mycoplasma‐free cells. All experiments in this study were approved by the Institutional Committee for Stem Cell Research and the Institutional Ethics Committee (Approval Reference No. RES2022062BAS).

iPSCs were routinely cultured in Essential 8 (E8) medium (Thermo Fisher Scientific) on Geltrex™‐coated plates (0.5 µg/cm^2^; Thermo Fisher Scientific) for the D149 line or Vitronectin‐coated plates (0.5 µg/cm^2^; Thermo Fisher Scientific) for the D2C and D3C lines. Cells were passaged at a 1:4 ratio upon reaching confluence using Accutase (Thermo Fisher Scientific) for D149 or 0.5 mM EDTA solution (Thermo Fisher Scientific) for D2C and D3C. Cells were then seeded onto freshly coated plates in E8 medium supplemented with 10 µM Y‐27632 (Sigma‐Aldrich).

### 2.2 RPE Differentiation

iPSCs were differentiated into iRPE cells using a previously established in‐house protocol [6, 7]. Briefly, confluent iPSCs maintained in E8 medium were transitioned to HUESC medium consisting of Advanced DMEM/F12 supplemented with 10% KnockOut™ Serum Replacement, 1× GlutaMAX™, 0.1 mM 2‐mercaptoethanol, and 1% penicillin–streptomycin (all from Thermo Fisher Scientific). Cells were sequentially directed through ectodermal, retinal, and RPE lineage stages.

For ectodermal induction, cells were cultured for 7 days in HUESC medium supplemented with 10 mM nicotinamide (Sigma‐Aldrich) and 1× N2 supplement (Thermo Fisher Scientific). Retinal lineage induction was subsequently performed by culturing cells in HUESC medium supplemented with 100 ng/mL Activin A (Thermo Fisher Scientific) for an additional 7 days. RPE maturation was achieved by maintaining the cells in HUESC medium for a further 4 weeks, until the appearance of characteristic pigmented iRPE patches.

To evaluate the effect of signalling pathway modulation on RPE differentiation efficiency, small molecules—including a Wnt activator, CHIR99021 (CHIR; 3 µM; Sigma), an FGF inhibitor, PD173074 (Fi; 1 µM; Sigma), and a MEK inhibitor, PD0325901 (Mi; 2 µM; Sigma)—were added during the maturation phase (day 14 to day 28). Cultures maintained in HUESC medium without small‐molecule supplementation served as controls. Differentiation efficiency was assessed based on pigmentation and MITF expression (flow cytometry). A diagrammatic representation of experimental design is provided in Figure S1.

### 2.3 Percentage Area Covered by Pigmented Patches

To assess differentiation efficiency, the percentage of the culture area covered by pigmented cell patches was quantified using image analysis. Briefly, following differentiation, images of the differentiation plates were captured. The area occupied by pigmented patches was measured using ImageJ/Fiji software and expressed as a percentage of the total culture area. Triplicate wells were analysed for each condition, and data are presented as the mean ± standard deviation. Statistical analysis was performed using one‐way ANOVA with Tukey's multiple‐comparisons correction, and differences were considered statistically significant at *p* < 0.001.

### 2.4 Flowcytometry Analysis

To evaluate differentiation efficiency, flow cytometry was performed to quantify the percentage of MITF‐positive cells in differentiated cultures. Briefly, following differentiation, cells were trypsinized, fixed with 4% paraformaldehyde, and permeabilized using 0.1% Triton X‐100. Samples were incubated with an anti‐MITF antibody for 1 h at room temperature, followed by incubation with secondary antibody (anti‐mouse 647) for 30 min at room temperature. Details of the antibodies used are provided in Table S1. Stained cells were washed and analysed using a BD FACSAria™ III flow cytometer, and data were processed using FlowJo software (BD Biosciences). Three independent replicates per condition were analysed for each cell line, and results are presented as the percentage of MITF‐positive cells (mean ± standard deviation). Statistical analysis was performed using two‐way ANOVA with Sidak's multiple‐comparisons correction, and differences were considered statistically significant at *p* < 0.0001.

### 2.5 iRPE Culture

For analysis of the effects of CHIR99021 treatment, iRPE cells derived from D149 iPSCs were used. Following differentiation, pigmented patches were manually isolated, enriched, and expanded in culture. The generated iRPE cells were maintained in RPE culture medium consisting of Advanced DMEM/F12 supplemented with 1× GlutaMAX, 1% penicillin–streptomycin, 10 mM nicotinamide, and 1% foetal bovine serum (all from Thermo Fisher Scientific), and cultured in an incubator (Heracell™ 150i, Thermo Fisher Scientific) maintained at 37°C with 5% CO_2_ and 95% humidity. Cells were routinely cultured on Geltrex™‐coated plates, with medium replaced twice weekly, and were passaged onto freshly coated plates every 2 weeks at a 1:4 split ratio. Serial passaging was continued up to passage 5 (P5).

To evaluate the effect of CHIR99021 treatment during iRPE maturation and serial passaging, CHIR99021 (CHIR; 3 µM; Sigma) was supplied continuously with the RPE culture medium throughout the experimental period. Control cultures were maintained in parallel without CHIR99021 supplementation. The medium was replaced twice weekly, with CHIR99021 included in the medium at each replacement. At each passage, cells were maintained under the respective treatment conditions and passaged at a 1:4 ratio every 2 weeks. Cultures were serially passaged up to P5, and at each experimental passage, cells were maintained under the respective treatment conditions for 1 month before analysis. A diagrammatic representation of experimental design is provided in Figure S1.

### 2.6 Quantitative Analysis of *LIN28A* as a Marker Associated With Residual Pluripotent Cells

To assess changes in expression of a marker associated with residual undifferentiated iPSC cells during prolonged iRPE culture, *LIN28A* expression was quantified by quantitative PCR (qPCR) across multiple iRPE passages following CHIR99021 treatment. qPCR was performed using a previously reported TaqMan‐based approach for *LIN28A* [8]. Total RNA was isolated from CHIR99021‐treated iRPE cells at different passages, as well as from undifferentiated hiPSCs and human primary RPE (hRPE) cells, which served as positive and negative controls, respectively. RNA extraction was performed using NucleoSpin RNA columns (Macherey‐Nagel), followed by cDNA synthesis using the High‐Capacity cDNA Reverse Transcription Kit (Applied Biosystems). qPCR was carried out using TaqMan™ Fast Advanced Master Mix with *LIN28A* (Cat No. 4453320, Assay ID: Hs00702808_s1; Applied Biosystems) and GAPDH TaqMan probes on a QuantStudio™ 5 Real‐Time PCR System (Applied Biosystems). *LIN28A* expression was normalized to GAPDH, and relative expression (RQ) values were calculated with reference to hRPE samples using Qbase+ software. Three independent replicates per condition were analysed, and data are presented as mean ± standard deviation. Statistical significance was determined using one‐way ANOVA with Tukey's multiple‐comparisons correction (*p* < 0.0001).

### 2.7 Estimation of Relative Optical Pigmentation

A melanin reference slide was prepared for normalization by dissolving synthetic melanin (16 mg/mL; Sigma) in DMSO, filtering through a 0.44 µm filter, and diluting in acetone and clear nail polish (1:1, v/v) to a final concentration of 1 mg/mL. Glass coverslips were uniformly coated with 500 µL of the melanin solution, dried for 30 s in a slanted position, and excess solution was gently removed; blank control slides were prepared without melanin. Relative optical pigmentation of iRPE cells at different passages, with or without CHIR99021 treatment, was quantified using a previously described method [9] with minor modifications to enable normalization to an optical reference standard and facilitate reliable comparison across imaging sessions.

Bright‐field images of iRPE cells were acquired at 10× magnification under constant illumination and camera gain settings using an inverted microscope (Eclipse TS100, Nikon). For each condition, three wells were analysed, with three fields imaged per well. To account for inter‐session imaging variability, blank and reference images were acquired in parallel during each imaging session. Relative optical pigmentation values were normalized to the corresponding reference image obtained under identical microscope settings and expressed as a percentage of the standard. Image processing and pigmentation quantification were performed using an ImageJ/Fiji plugin script (Supplementary file 1). A minimum of three independent replicates per condition were analysed and results are presented as percentage relative optical pigmentation (mean ± standard deviation). Statistical analysis was performed using one‐way ANOVA with Tukey's multiple‐comparisons correction, and differences were considered statistically significant at *p* < 0.05.

### 2.8 Quantitative PCR

Total RNA was isolated using the NucleoSpin RNA Kit (Macherey‐Nagel) according to the manufacturer's instructions, and RNA concentration and purity were assessed using a spectrophotometer (NanoDrop™ 1000, Thermo Fisher Scientific). cDNA was synthesized using the High‐Capacity cDNA Reverse Transcription Kit (Applied Biosystems). qPCR was performed using TB Green® Master Mix (Takara Bio) on a QuantStudio™ 5 Real‐Time PCR System (Applied Biosystems) with custom primers (Integrated DNA Technologies; listed in Table S2). Gene expression was normalized to β‐ACTIN and GAPDH, which were identified as stable reference genes using GeNorm analysis (Figure S2). Relative gene expression levels were calculated using Qbase+ software. Normalized relative quantities (RQ) were used for data visualization, and statistical significance was assessed using one‐way ANOVA followed by Tukey's multiple‐comparisons test (*p* ≤ 0.05).

### 2.9 Immunostaining

Cells were fixed with 4% paraformaldehyde (HiMedia) for 20 min at room temperature and washed with PBS. The cells were then permeabilized with 0.1% Triton X‐100 (Sigma‐Aldrich) for 5 min at room temperature and rinsed with PBS. Blocking was performed using 2% bovine serum albumin (BSA; Sigma‐Aldrich) for 10 min. Samples were incubated for 2 h at room temperature with the primary antibody. After washing with PBS, cells were incubated with the appropriate secondary antibody for 1.5 h at room temperature in the dark. Details of the antibodies used are provided in Table S1. Nuclei were counterstained with 0.1 µg/mL DAPI (Sigma‐Aldrich) for 2 min. Images were acquired using a Leica Stellaris STED confocal microscope equipped with a 63× oil immersion objective and analysed using Fiji (ImageJ).

### 2.10 Western Blotting

Proteins were extracted using ice‐cold RIPA lysis buffer (HiMedia) followed by sonication, and protein concentration was determined using the DC Protein Assay Kit (Biorad). Equal amounts of protein were denatured at 95°C for 5 min and separated by SDS–PAGE using a 5% stacking gel with either 10% resolving gels for MERTK and Na^+^/K^+^‐ATPase or a 12.5% resolving gel for SLC16A8. Proteins were transferred onto PVDF membranes (0.4 μm for MERTK and Na^+^/K^+^‐ATPase; 0.2 μm for SLC16A8), and transfer efficiency was confirmed using the Pierce™ Reversible Protein Stain Kit (Thermo Fisher Scientific). Membranes were blocked in 5% BSA or 5% milk prepared in TBST and incubated with primary antibodies against GAPDH, MERTK, Na^+^/K^+^‐ATPase, and SLC16A8, followed by HRP‐conjugated secondary antibodies. Details of the antibodies used are provided in Table S1. Protein bands were detected using enhanced chemiluminescence (Thermo Fisher Scientific) and imaged using a Bio‐Rad Gel Doc system. Band intensities were quantified using ImageJ software and normalized to GAPDH.

### 2.11 RNA Sequencing

Total RNA was isolated from passage 4 (P4) iRPE cells derived from the D149 iPSC line from the control and CHIR99021‐treated groups using the NucleoSpin RNA Kit (Macherey–Nagel), according to the manufacturer's instructions. RNA quality and quantity were assessed using the Agilent TapeStation 4150 and Qubit High Sensitivity DNA Assay. All RNA samples used for sequencing had an RNA Integrity Number (RIN) greater than 8, with RIN values ranging from 8.8 to 9.1.

The RNA‐sequencing experiment included three independent biological replicates per condition (control, *n* = 3; CHIR99021‐treated, *n* = 3), with each replicate generated through an independent iRPE differentiation using the same iPSC line (D149). Samples from different passages were not pooled, and all samples were analysed individually. Control and CHIR99021‐treated samples were processed in parallel using the same RNA extraction, library preparation, and sequencing procedures. mRNA libraries were prepared, and RNA sequencing was performed on the Illumina NovaSeq. 6000 platform. Sequencing generated approximately 9.0–20.4 Gb of data per sample, corresponding to approximately 29.4–66.6 million paired‐end reads after adapter trimming. No evidence of a batch effect was observed during the RNA‐seq analysis; therefore, batch correction was not applied.

Raw sequencing reads were subjected to quality control to assess base quality distribution, GC content, and adapter contamination. Low‐quality reads and adapter sequences were trimmed using Cutadapt (v3.5), and only high‐quality reads were retained for downstream analysis. The filtered paired‐end reads were aligned to the human reference genome (GRCh38) using the splice‐aware aligner STAR (v2.7.11b). Alignment quality was evaluated, with uniquely mapped reads ranging between ~89–91% across samples. Gene‐level read counts were generated using Feature Counts (v2.0.1) and subsequently normalized using the DESeq. 2 package in R. Principal component analysis (PCA) and hierarchical clustering were performed in R to assess sample relationships and data variability. Differential gene expression analysis was conducted using DESeq. 2, with genes exhibiting an adjusted *p*‐value ≤ 0.05 and log2 fold change ≥ 2 considered significantly differentially expressed. Functional enrichment analysis was performed using Gene Set Enrichment Analysis (GSEA) implemented in the clusterProfiler R package, interrogating Gene Ontology (GO), Reactome, and Hallmark gene sets. Pathways with a nominal *p*‐value < 0.05 were considered significantly enriched.

## 3 Results

### 3.1 Modulation of Wnt Signalling Enhances iPSC Differentiation toward the RPE Lineage

Wnt/β‐catenin, FGF, and MAPK signalling pathways have important roles in RPE development and differentiation [10, 11]. To determine whether modulation of these pathways influence RPE differentiation, iPSCs were treated during the final 14 days of differentiation with CHIR99021 (GSK‐3α/β inhibitor commonly used to modulate Wnt/β‐catenin signalling), PD173074 (FGF inhibitor), or PD0325901 (MEK inhibitor), alone or in combination. Differentiation efficiency was assessed by quantifying pigmented areas. CHIR99021 treatment significantly increased pigmentated area compared with untreated controls, FGF inhibition, or MEK inhibition (Figure 1a,b). The CHIR99021 + PD0325901 combination showed the largest pigmented area numerically; however, the difference in pigmented area was not statistically significant compared with CHIR99021 treatment alone. Flow cytometric analysis of MITF expression confirmed enhanced RPE differentiation following CHIR99021 treatment, with CHIR99021‐treated cultures exhibiting the highest proportion of MITF‐positive cells (91%) compared with untreated controls (50%) (Figure 1c). Similar trends were observed across three independent iPSC lines (Figure 1d). Together, These findings support CHIR99021 as an effective treatment condition among those tested for promoting the efficiency of iRPE differentiation from iPSCs.

**Figure 1:**
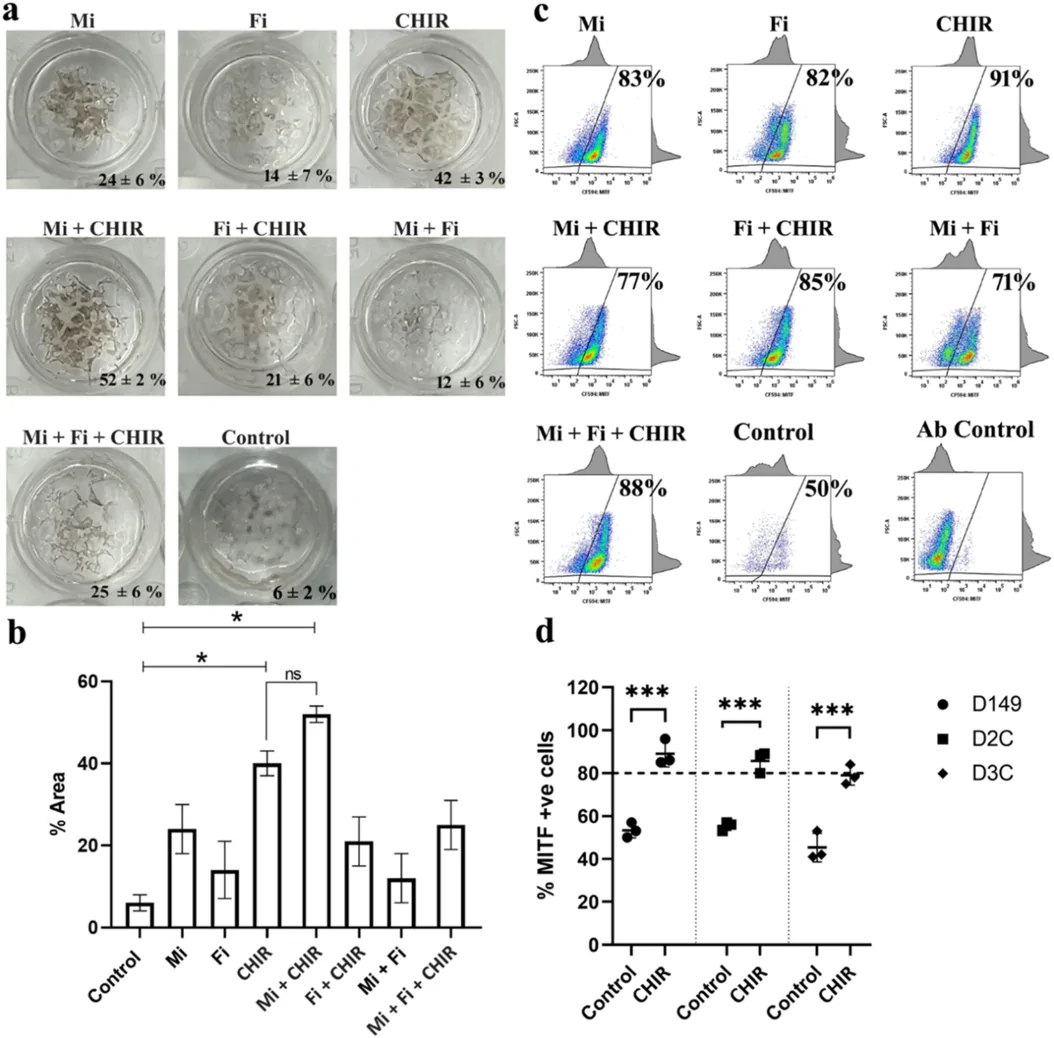
Small‐molecule screening identifies CHIR99021 as an effective treatment for promoting iRPE generation during iPSC differentiation. (a) Representative images of culture wells showing the extent of pigmentation under the indicated treatment conditions. Values shown below each image represent the percentage area (mean ± standard deviation) occupied by pigmented cellular patches. (b) Quantification of the percentage area of pigmented patches under control conditions (no small‐molecule treatment) and following small‐molecule treatments. Statistically significant differences are indicated by * (*p* < 0.001), as determined by one‐way ANOVA with Tukey's multiple‐comparisons correction. (c) Flow‐cytometric analysis of MITF expression under different treatment conditions. Inset values indicate the percentage of MITF‐positive cells. (d) Quantification of MITF‐positive cells from three independent iPSC lines (D149, D2C, and D3C) under control conditions (no small‐molecule treatment) and following CHIR99021 treatment (CHIR; CHIR99021). Statistically significant differences are indicated by *** (*p* < 0.0001), as determined by two‐way ANOVA with Sidak's multiple‐comparisons correction. Abbreviations: Mi, MEK pathway inhibitor PD0325901; Fi, FGF pathway inhibitor PD173074; CHIR, GSK3α/β inhibitor CHIR99021. Combination treatments are denoted as Mi+CHIR, Fi+CHIR, Mi+Fi, and Mi+Fi+CHIR.

The differentiation approach used in this study was based on our previously established and reported iRPE differentiation protocol [6, 7, 9]. In our previous studies, pigmented patches generated during differentiation were isolated and expanded, and the resulting cells were characterized for RPE‐associated marker expression and cellular features and subsequently used for disease modelling. In the present study, we modified this established approach by introducing small‐molecule treatments during the final maturation phase (day 14 to day 28) to investigate whether the efficiency of iRPE generation could be improved. Therefore, rather than repeating an extensive characterization of the differentiated cells, we used the extent of pigmented areas as a morphological measure of pigment‐positive iRPE generation. In addition, MITF expression was assessed by flow cytometry as an independent molecular indicator of the RPE phenotype.

### 3.2 CHIR99021 Treatment Reduces *LIN28A* Expression in iRPE Cultures

Residual pluripotent or partially differentiated cells remain a persistent concern in iPSC‐derived cultures [12], as even low levels of such cells may compromise experimental reproducibility and limit translational applications. To determine whether CHIR99021 treatment influences the expression of a pluripotency‐associated marker during iRPE culture, *LIN28A* expression was examined across multiple passages. *LIN28A* was selected based on a previously reported TaqMan‐based qPCR approach for detecting residual undifferentiated hiPSCs in iPSC‐derived RPE cultures. Kuroda et al [8] reported that *LIN28A* showed greater sensitivity than several other pluripotency‐associated markers for detecting trace levels of undifferentiated hiPSCs in RPE cultures, with detection of approximately 0.002% residual hiPSCs. In our study, undifferentiated iPSCs showed high *LIN28A* expression, whereas primary RPE cells showed minimal expression. Although passage 0 iRPE cultures retained detectable *LIN28A* expression, CHIR99021 treatment was associated with reduced *LIN28A* expression during subsequent passaging. *LIN28A* levels in P0, P2, P4, and P7 iRPE cultures were not significantly different from those observed in primary hRPE cells, whereas all iRPE groups showed significantly lower *LIN28A* expression than undifferentiated iPSCs (Figure S3). The reduced *LIN28A* expression observed in CHIR99021‐treated cultures is consistent with reduced persistence of cells expressing this pluripotency‐associated marker and may suggest a reduction in residual undifferentiated cell populations. However, to conclusively establish the elimination of residual pluripotent cells, further assessment using functional assays would be required.

### 3.3 CHIR99021 Treatment Enhances Relative Optical Pigmentation During iRPE Maturation

Having established the effect of CHIR99021 treatment during iRPE differentiation, we next sought to semi‐quantitatively assess pigmentation dynamics during the subsequent culture. To facilitate standardized and reproducible pigmentation measurements across time points and imaging sessions, we developed a custom ImageJ/Fiji plugin (script provided in Supplementary File 1) that streamlines image analysis and data extraction. The overall workflow for relative optical pigmentation quantification, including representative bright‐field images, image inversion, and signal extraction steps, is illustrated in Figure S4. Briefly, bright‐field images in TIFF format were analysed using the plugin, which performs grayscale conversion, image inversion, blank correction, and integrated density measurements in a batch‐processing format. The resulting relative optical pigmentation signals were subsequently normalized to optical reference to calculate percentage pigmentation values. This workflow enabled reliable comparison of pigmentation levels across culture stages and treatment conditions.

Using this approach, iRPE cultures maintained in the presence of CHIR99021 exhibited significantly greater relative optical pigmentation than untreated controls throughout the culture period (Figure 2a). These findings indicate that CHIR99021 treatment promotes pigment accumulation during iRPE maturation.

**Figure 2:**
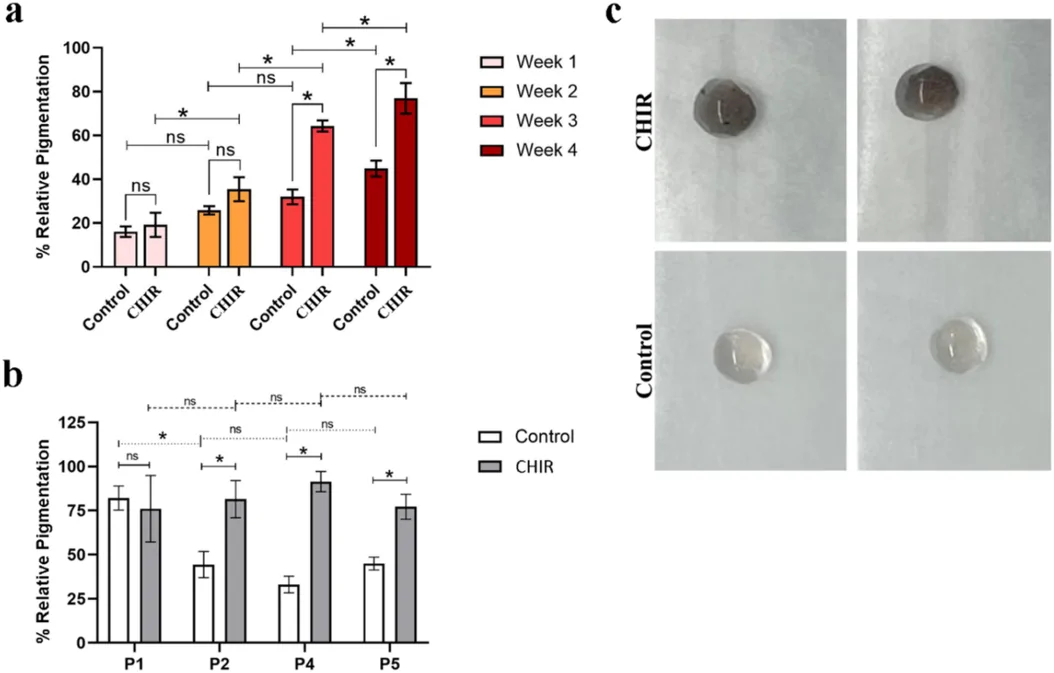
CHIR99021 treatment enhances relative optical pigmentation during iRPE culture and preserves pigmentation after passaging. (a) Semi‐quantitative analysis showing increased relative optical pigmentation in CHIR99021(CHIR)‐treated iRPE cultures compared with controls over the 1‐month culture maturation period. Data represent mean ± standard deviation from three independent biological replicates (*p* < 0.05; two‐way ANOVA with Tukey's correction). (b) Relative optical pigmentation following passaging (P1, P2, P4, and P5). While relative optical pigmentation was comparable at P1, CHIR99021(CHIR)‐treated iRPE cultures retained significantly greater relative optical pigmentation at later passages, whereas control cultures showed a progressive reduction in relative optical pigmentation (*p* < 0.05; one‐way ANOVA with Tukey's correction). (c) Representative image of membrane inserts (cut cell inserts with PBS added on top) showing iRPE cells (passage 4, 4‐week‐old cultures) maintained either with CHIR99021 (CHIR) or without CHIR99021 (Control).

### 3.4 CHIR99021 Treatment Preserves iRPE Relative Optical Pigmentation After Passaging

iRPE cells typically exhibit a gradual loss of pigmentation and other RPE‐specific characteristics upon passaging. To determine whether CHIR99021 treatment could mitigate this decline, iRPE cultures were passaged in the presence of the CHIR99021 and compared with untreated controls. Semi‐quantitative relative optical pigmentation analysis revealed that CHIR99021‐treated cells retained robust pigmentation following passaging, whereas untreated cultures showed a marked reduction in relative optical pigmentation (Figures 2b,c, S5). These findings indicate that CHIR99021 treatment is associated with preservation of relative optical pigmentation during passaging.

### 3.5 CHIR99021 Treatment Drives Distinct Transcriptional Changes in iRPE Cells

To investigate the effects of CHIR99021 treatment, RNA sequencing was performed on passage 4 iRPE cells continuously exposed to CHIR99021 from differentiation through passage 4 and compared with passage‐matched untreated controls. Quality control analyses showed clear separation between CHIR99021‐treated and control samples, with high consistency among biological replicates based on hierarchical clustering and sample‐to‐sample correlation analyses (Figure S6a,b). Differential expression analysis identified distinct sets of significantly upregulated and downregulated genes in response to CHIR99021 treatment (Figure S6c), indicating that CHIR99021 treatment induces substantial transcriptional remodelling in iRPE cells.

### 3.6 CHIR99021 Treatment Is Associated with Coordinated Transcriptional Remodelling

Gene Ontology (GO) enrichment analysis revealed coordinated transcriptional changes following CHIR99021 treatment (Figure 3). Enriched cellular component terms included ribosomal, pigment granule, endoplasmic reticulum, lysosomal, and transport‐associated components (Figure 3a). Biological process analysis showed enrichment of translation, ribonucleoprotein biogenesis, pigment metabolism, and lysosome‐related pathways, accompanied by suppression of cell‐cycle‐associated processes (Figure 3b). Molecular function analysis further identified enrichment of ribosomal, ion transport, lipid metabolism, and signalling‐related functions, together with reduced actin‐binding activity (Figure 3c). These findings indicate changes in transcriptional programmes associated with biosynthetic activity, pigmentation, lysosomal processes, and cell cycle regulation. Hallmark gene set enrichment analysis further identified enrichment of NOTCH, mTORC1, unfolded protein response, TGF‐β signalling, and apical junction‐associated gene sets, together with suppression of E2F targets, G2M checkpoint, and mitotic spindle pathways (Figure S7a). Interferon response pathways were modestly enriched, whereas inflammatory response pathways were reduced. Reactome analysis similarly demonstrated enrichment of RNA processing, translational initiation, and protein targeting pathways, alongside downregulation of cell‐cycle‐related pathways (Figure S7b). Together, these analyses demonstrate coordinated transcriptional remodelling following CHIR99021 treatment, including changes in programmes associated with pigmentation, lysosomal activity, epithelial organization, biosynthetic processes, and cell‐cycle regulation.

**Figure 3:**
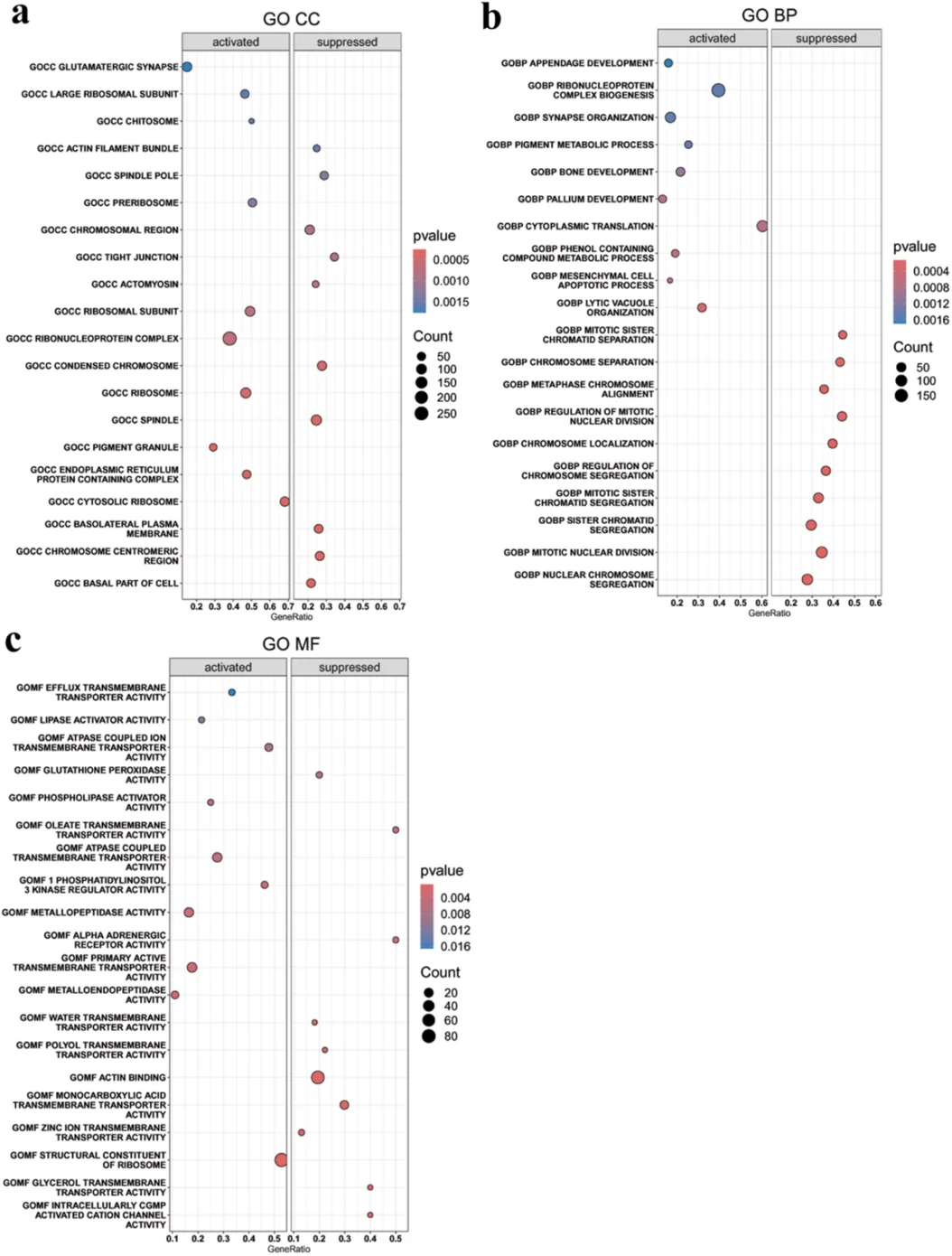
Gene Ontology enrichment analysis of differentially expressed genes in CHIR99021 treated iRPE cells. Bubble plots show significantly enriched GO terms (FDR < 0.05) among differentially expressed genes. A total of 417 upregulated genes (log_2_ fold change ≥ 2) and 794 downregulated genes (log_2_ fold change ≤ −2) were included in the analysis. (a) GO‐CC highlights ribosome‐ and endoplasmic reticulum–associated components and pigment granules; (b) GO‐BP indicates increased cytoplasmic translation, pigment metabolism, and lysosome organization with reduced cell‐cycle processes; (c) GO‐MF shows enhanced ribosomal, ATPase‐coupled transport, lipid‐processing, and PI3K regulatory activities with diminished actin‐binding functions. Activated and supressed gene sets/pathways are indicated. Bubble size represents gene count and colour denotes statistical significance (*p*‐value).

### 3.7 GSEA Identifies Changes in Pigmentation‐ and Epithelial‐Associated Transcriptional Programmes Following CHIR99021 Treatment

Gene set enrichment analysis (GSEA) revealed significant changes in transcriptional programmes associated with pigmentation and epithelial organization in CHIR99021‐treated cells compared with controls (Figure 4). Positively enriched pathways included pigmentation‐ and MITF‐related programs, together with gene sets associated with cellular transport, epithelial junction organisation, extracellular matrix interactions, and blood–retinal barrier‐associated processes. In contrast, pathways associated with pluripotency, cell‐cycle progression, and MHC class II antigen presentation were negatively enriched. These findings indicate that CHIR99021 treatment is associated with changes in transcriptional programs related to pigmentation, epithelial organization, and cellular state, together with reduced proliferation‐ and stemness‐associated programmes.

**Figure 4:**
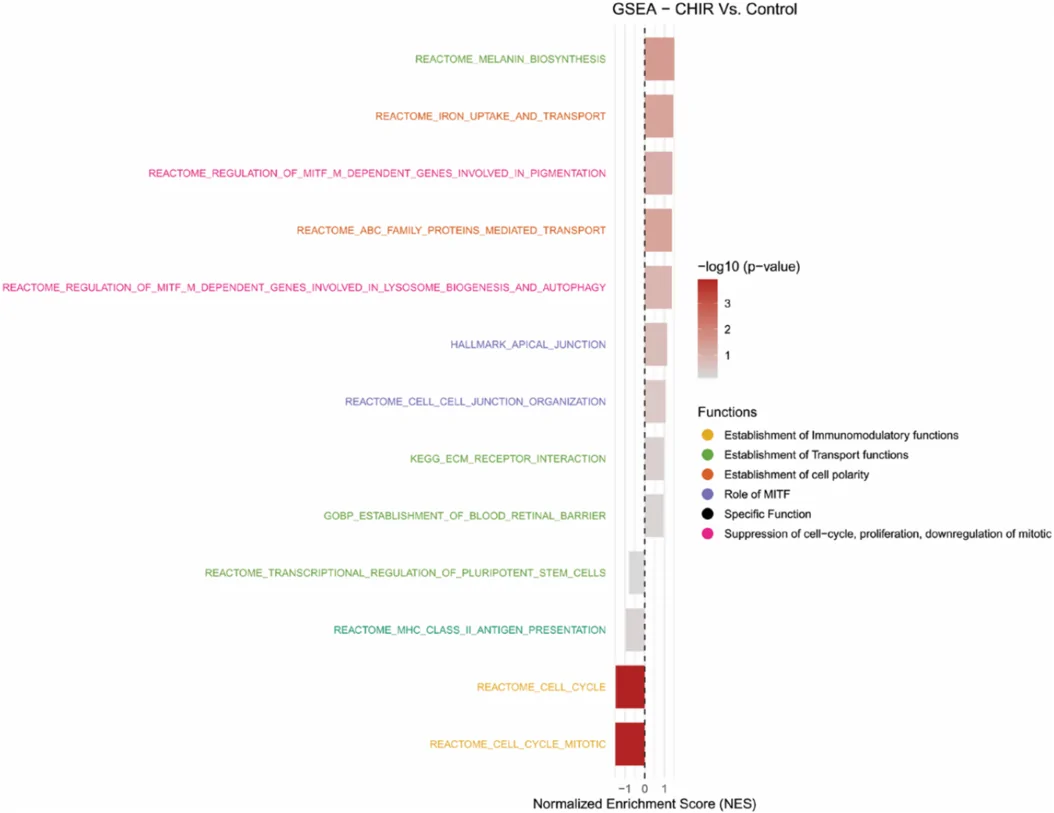
Gene Set Enrichment Analysis of CHIR99021‐treated iRPE cells. Gene Set Enrichment Analysis (GSEA) comparing CHIR99021‐treated and control iRPE cells reveals pathway‐level transcriptional changes following CHIR99021 treatment. Enrichment plots depict genes ranked by differential expression between CHIR and control groups, with normalized enrichment scores (NES) scaled from −1 to +1 to indicate the direction and relative magnitude of pathway regulation. Colour intensity represents −log10 (P value) for pathway enrichment.

### 3.8 CHIR99021 Treatment Reduced RPE Functional Gene Expression

Analysis of visual cycle genes revealed distinct transcriptional changes in CHIR99021‐treated iRPE cells (Figure 5a). Genes involved in retinol metabolism and transport were generally upregulated, whereas key visual cycle genes, including RPE65 and LRAT, were downregulated (Figure S8). Reduced RPE65 expression was further confirmed by qPCR at higher passages (Figure S9), indicating that CHIR99021 treatment enhances aspects of retinoid metabolism while suppressing components of the canonical visual cycle.

**Figure 5:**
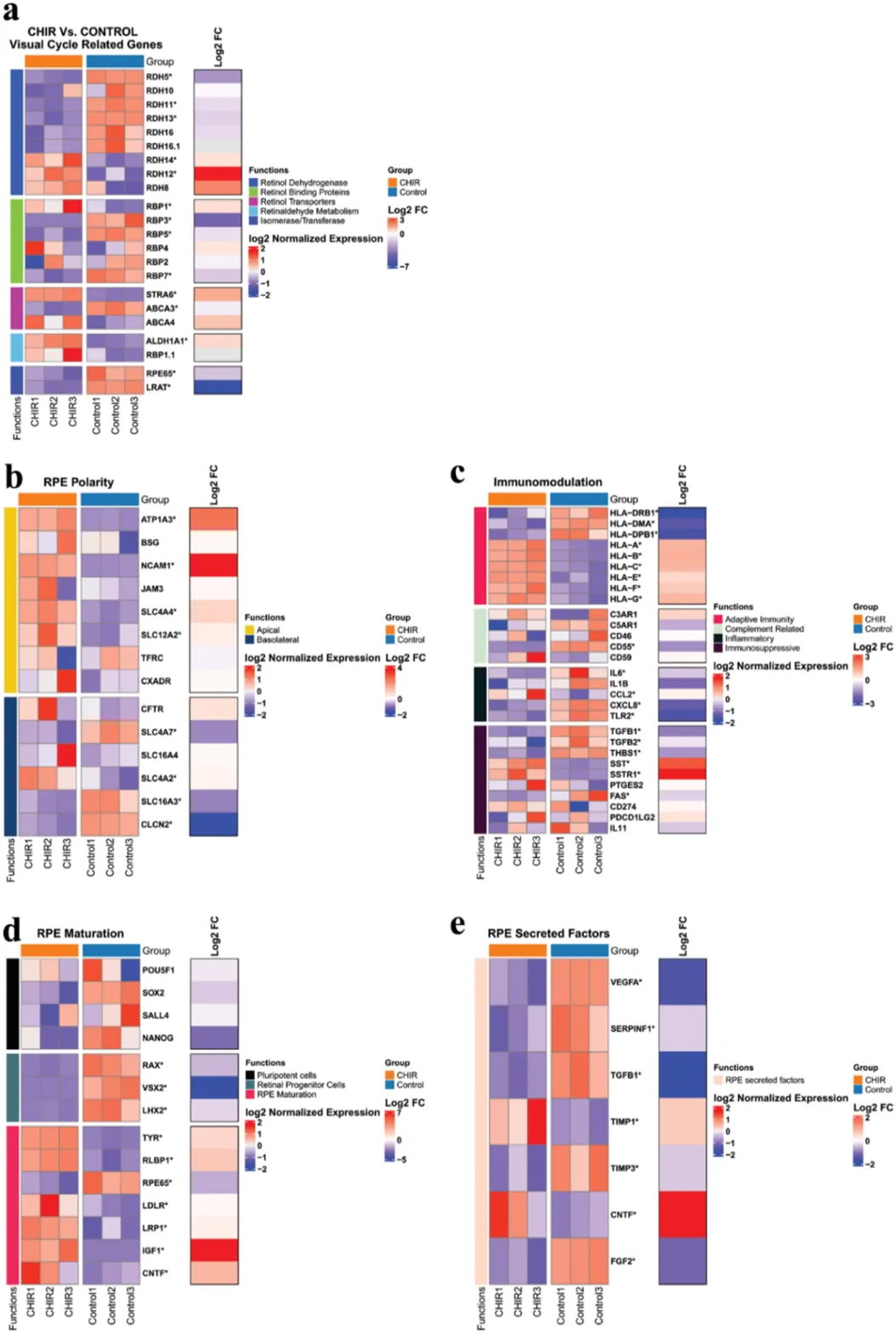
CHIR99021 treatment alters gene expression associated with the visual cycle, polarity, maturation, immunomodulation, and secretory functions in iRPE cells. (a) Heatmap showing normalized expression and fold change of selected visual cycle and retinoid metabolism genes in control and CHIR‐treated iRPE cells. Several retinoid metabolism–related genes exhibit altered expression following CHIR99021 treatment. (b) Heatmap of genes associated with RPE polarity, indicating transcriptional changes linked to epithelial organization and polarity maintenance. (c) Heatmap of genes involved in RPE immunomodulatory functions, showing altered immune regulatory and inflammatory signalling following CHIR treatment. (d) Heatmap of genes associated with RPE maturation and functional identity, demonstrating changes in maturation‐related transcriptional programs. (e) Heatmap of genes encoding RPE‐secreted factors involved in retinal homeostasis, indicating modulation of the secretory profile upon CHIR99021 treatment. For all panels, rows represent selected genes and columns represent biological replicates from control and CHIR‐treated groups. Heatmaps depict normalized expression levels, with adjacent bars showing log fold change (Log FC). Asterisks (*) indicate significantly altered genes.

Examination of polarity‐associated genes showed increased expression of several apical transport and adhesion molecules, including ATP1A3, NCAM1, and SLC4A4, together with reduced expression of selected basolateral markers (Figure 5b), consistent with enhanced epithelial polarization. Analysis of immunomodulatory genes revealed broad remodelling of immune‐related pathways, characterized by reduced expression of MHC class II genes, complement regulators, and inflammatory mediators such as IL6, CXCL8, and TLR2, alongside increased expression of MHC class I genes (Figure 5c).

Several markers associated with RPE maturation, including TYR, RLBP1, LDLR, LRP1, IGF1, and CNTF, were upregulated in CHIR99021‐treated cells, whereas pluripotency‐ and retinal progenitor‐associated genes showed reduced expression (Figure 5d). qPCR analysis demonstrated similar trends for selected markers (Figure S9). However, RPE65 remained significantly downregulated, indicating incomplete acquisition of specialized RPE functions. Analysis of secreted factors further revealed altered expression of genes involved in angiogenesis, extracellular matrix regulation, immune signalling, and neurotrophic support (Figure 5e). In addition, altered aquaporin expression suggested modulation of epithelial transport pathways (Figure S10). Consistent with these findings, GSEA revealed suppression of transcriptional programs associated with microvillus assembly, phagocytosis, and cilium‐related pathways in CHIR99021‐treated iRPE cells (Figure 6). Reduced Ezrin expression, despite enhanced F‐actin localization at cell borders and decreased cellular size, were observed following CHIR99021 treatment (Figure S11). Collectively, these findings indicate that CHIR99021 treatment is associated with some changes in epithelial‐associated transcriptional programs and pigmentation, while not uniformly supporting transcriptional programs associated with specialized RPE functions, including visual‐cycle, phagocytic, and ciliary processes.

**Figure 6:**
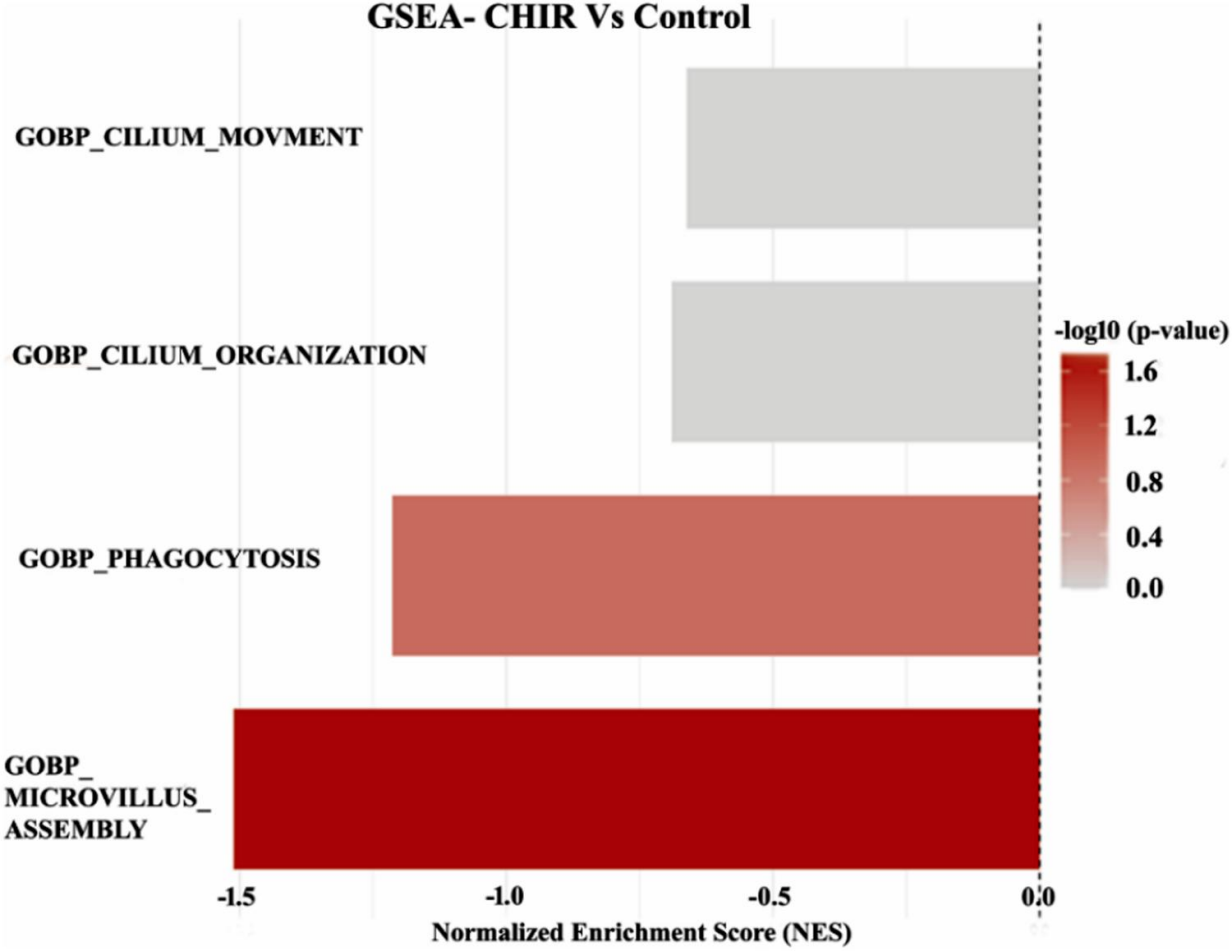
Gene set enrichment analysis reveals suppression of microvillus assembly, phagocytosis, and cilium‐related pathways in CHIR99021 treated iRPE cells. Gene set enrichment analysis (GSEA) comparing CHIR‐treated and control iRPE cells reveals pathway‐level transcriptional changes associated with CHIR99021 treatment. The analysis highlights differential regulation of pathways related to microvillus assembly, phagocytosis, and cilium organization, suggesting that CHIR99021 treatment affects structural and functional programs associated with RPE polarity and photoreceptor‐supporting functions. Bars represent the normalized enrichment score (NES) for selected Gene Ontology biological processes, indicating the direction and magnitude of pathway regulation in CHIR‐treated cells relative to controls. Negative NES values indicate pathways enriched in control cells, whereas positive NES values indicate enrichment in CHIR‐treated cells. Colour intensity represents −log10(P value), reflecting the statistical significance of pathway enrichment.

### 3.9 CHIR99021 Treatment Is Associated With Reduced Expression of RPE‐Associated Transcriptional Programme

To further examine whether the enhanced pigmentation observed following CHIR99021 treatment was accompanied by broader RPE‐associated transcriptional changes, we analysed the DESCARTES_MAIN_FETAL_RETINAL_PIGMENT_CELLS gene set from MSigDB, comprising 265 genes associated with foetal retinal pigment cells. Analysis of the normalized expression data identified 113 genes for which differential‐expression values were available. Of these, 77 genes showed negative log_2_ fold changes and 36 showed positive log_2_ fold changes in CHIR99021‐treated cells compared with controls, with 66 genes significantly downregulated and 25 significantly upregulated (*p* < 0.05).

Based on a raw count threshold of > 20, 148 genes were not detected in either condition, while 104 genes were detected in CHIR99021‐treated cells and 105 in control cells, with 101 genes detected in both conditions. A supervised heatmap of the normalized expression of these 101 genes further illustrated the predominance of lower expression across many genes in CHIR99021‐treated cells compared with controls (Figure S12). Consistent with these observations, GSEA demonstrated significant negative enrichment of the DESCARTES_MAIN_FETAL_RETINAL_PIGMENT_CELLS gene set in CHIR99021‐treated cells compared with controls (Figure S13).

These findings indicate that, although CHIR99021 treatment enhances pigmentation and is associated with epithelial‐associated features, it does not uniformly enhance broader RPE‐associated transcriptional programs, suggesting that increased pigmentation and epithelial‐associated features do not necessarily reflect broader transcriptional maturation of the RPE phenotype.

## 4 Discussion

Pigmentation is a defining hallmark of RPE and reflects melanosome biogenesis and melanin homeostasis. RPE melanosomes arise during early development and predominantly contain eumelanin, which contributes to cellular homeostasis through absorption of excess light, antioxidant activity, and protection against photo‐oxidative stress [13, 14]. Although melanogenesis largely stabilizes after birth, maintenance of pigmentation remains an important characteristic of RPE cells, and melanin deficiency has been associated with increased susceptibility to oxidative stress and light‐induced cellular damage [15, 16, 17, 18]. However, it remains unclear whether pigmentation alone can serve as an indicator of the acquisition of specialized RPE functions. Despite its widespread use as an indicator of RPE differentiation, the relationship between pigmentation and broader aspects of functional maturation remains incompletely understood. In this study, iRPE cells were serially passaged and matured to examine whether pigmentation and RPE‐associated characteristics were retained during prolonged culture with CHIR99021 treatment. Establishing culture conditions that maintain the phenotypic and functional characteristics of RPE cells during extended culture could be valuable for generating sufficient numbers of cells for various downstream applications [19].

Wnt/β‐catenin signalling has been reported to play an important role in early RPE specification during optic cup development [20, 21], with β‐catenin activation promoting the expression of RPE determinants such as MITF and OTX2 [22, 23]. Consistent with these observations [24, 25], CHIR99021 treatment in our system increased number of pigmented iRPE patches and was associated with a higher proportion of MITF‐positive cells. Wnt signalling is not restricted to early developmental stages. In the mature eye, Wnt ligands produced by the neural retina, lens epithelium, and RPE establish autocrine and paracrine signalling networks that contribute to epithelial maintenance and suppression of transdifferentiation [20, 21, 22]. Conversely, dysregulated Wnt activity has been associated with impaired differentiation, altered barrier properties, and increased susceptibility to cellular stress [26, 27], suggesting that appropriate regulation of Wnt signalling may be important for maintaining RPE identity.

CHIR99021, a GSK‐3α/β inhibitor [28], is commonly used for the pharmacological modulation of Wnt/β‐catenin signalling and has therefore been widely incorporated into stem cell differentiation protocols [24, 29]. However, most previous studies have focused on the role of Wnt signalling during early lineage specification, whereas the effects of CHIR99021 treatment on already differentiated RPE cells during prolonged culture remain less well understood. To further characterize the transcriptional response to CHIR99021 exposure, we examined Wnt‐associated genes and pathways in our RNA‐seq dataset. This analysis revealed substantial transcriptional remodelling of Wnt‐associated genes, including statistically significant upregulation of established Wnt‐responsive genes such as AXIN2, LEF1, NKD1, SP5, RNF43, ZNRF3, and FZD7, together with increased expression of other pathway‐associated genes, including CTNNB1, TCF7L1, and FZD1. Several genes associated with non‐canonical Wnt signalling, including WNT5A, WNT5B, ROR2, VANGL2, and DAAM1, were also significantly upregulated (Figure S14a). GSEA showed directional enrichment of the Hallmark Wnt/β‐catenin signalling gene set (Figure S14b) and the Reactome β‐catenin‐independent Wnt signalling gene set (Figure S14c), whereas the Reactome TCF‐dependent signalling in response to Wnt gene set showed a negative enrichment trend (Figure S14d). However, none of these gene‐set enrichments reached statistical significance. These findings indicate substantial transcriptional remodelling of Wnt‐associated genes following CHIR99021 treatment during prolonged iRPE culture, with directional enrichment of both β‐catenin‐associated and β‐catenin‐independent Wnt gene sets. However, the absence of statistically significant GSEA enrichment and the divergent behaviour of the TCF‐dependent Wnt‐response gene set preclude definitive conclusions regarding sustained activation of specific canonical or non‐canonical Wnt signalling programmes.

Although pigmentation is widely considered an indicator of RPE identity, its relationship to RPE functionality remains poorly defined. A major challenge in studying pigmentation dynamics is the ability to repeatedly assess relative pigmentation without disrupting the cultures. Although spectrophotometric [30, 31] and flow‐cytometry‐based [32] approaches provide quantitative melanin‐related measurements, both generally require cell harvesting, extraction, or dissociation and therefore do not allow repeated assessment of the same intact cultures. We previously developed a non‐ destructive bright‐field image–based method for assessing relative optical pigmentation [9]. However, direct comparison across imaging sessions using this approach was limited by variability in microscope illumination and acquisition settings. To address this limitation, we incorporated an optical reference that was imaged alongside experimental samples, enabling normalization of relative pigmentation measurements across time points and experimental conditions. This improved semi‐quantitative approach enabled repeated assessment of relative optical pigmentation during prolonged culture and serial passaging without disrupting the cultures. Importantly, the assay provides a measure of relative optical pigmentation rather than direct biochemical quantification of cellular melanin content; however, it is sufficient for monitoring relative changes in pigmentation over time. Using this approach, we demonstrate that CHIR99021 treatment enhances relative optical pigmentation and its retention during prolonged culture and serial passaging. While previous work has shown that spatially and temporally controlled Wnt stimulation can enhance the formation of pigmented RPE‐like regions in retinal organoids [33], our findings suggest that continued CHIR99021 treatment during the maintenance phase may further enhance the retention of the pigmented phenotype in differentiated iRPE cultures.

Although Wnt activation has previously been associated with proliferation in RPE cells [34, 35], CHIR99021 treatment in our system was associated with reduced cell‐cycle and retinal progenitor‐associated transcriptional programs together with changes in epithelial organization. Transcriptomic analysis showed suppression of cell‐cycle pathways and retinal progenitor markers, together with enrichment of pigmentation, lysosomal, metabolic, polarity‐associated, and epithelial barrier pathways. Loss of epithelial identity is a recognized limitation of long‐term iRPE cultures [36, 37] and has been characterized by loss of cobblestone morphology and emergence of fibroblast‐like cells. In contrast, CHIR99021‐treated cells showed enhanced localization of F‐actin to cell borders and reduced cellular and nuclear size (Figure S11), consistent with preservation or enhancement of epithelial organization rather than the morphological deterioration commonly reported in prolonged iRPE cultures. Interestingly, smaller RPE cells are characteristic of macular RPE [38, 39, 40], which also exhibits distinct lysosomal activity [41, 42], enhanced lipid metabolism, distinct angiogenic and inflammatory programs [43, 44], as well as lower expression of Na^+^/K^+^‐ATPase and monocarboxylate transporters compared with peripheral RPE [39, 45]. Consistent with these reported characteristics, CHIR99021‐treated iRPE cells showed enrichment of lysosomal and lipid metabolic pathways together with reduced expression of Na^+^/K^+^‐ATPase and SLC16A8/MCT3 (Figure S15). CHIR99021 treatment was also associated with changes in immune and secretory transcriptional programs, including downregulation of MHC class II genes, IL6, and CXCL8, as well as altered expression of secreted factors such as VEGFA, SERPINF1, TGFB1, TIMP3, and CNTF. Together, these findings suggest that CHIR99021 treatment was associated with pigmentation and epithelial features that overlap with characteristics reported in macular RPE. However, without direct protein‐level and functional validation, these associations do not establish enhanced epithelial, barrier, metabolic, immune, or secretory function.

Despite these changes, CHIR99021 treatment during prolonged iRPE culture did not uniformly enhance transcriptional programs associated with specialized RPE functions. Transcriptomic analyses demonstrated reduced expression of pathways related to cilium organization and phagocytosis. Consistent with these findings, immunostaining revealed reduced Ezrin‐positive microvillus structures (Figure S11), while western blot analysis showed decreased expression of MERTK (Figure S15), a receptor involved in photoreceptor outer segment phagocytosis and localized to the apical region of RPE cells. Moreover, key visual cycle enzymes, including RPE65 and LRAT, were downregulated. Further supporting this distinction, analysis of the DESCARTES foetal retinal pigment cell gene set revealed an overall tendency toward reduced expression of RPE‐associated genes following CHIR99021 treatment. These findings suggest that increased pigmentation following CHIR99021 treatment in prolonged iRPE culture does not necessarily correspond to a broader enhancement of RPE‐associated transcriptional programs.

These observations are consistent with a previous study showing that inhibition of canonical Wnt signalling using IWP2 and endo‐IWR1 promoted cell‐cycle exit, enhanced RPE65 expression, and improved formation of primary cilia and phagocytic activity compared with untreated or CHIR99021‐treated iRPE cells [46]. However, Wnt inhibition alone was insufficient to achieve complete RPE maturation and apical–basal polarization, which additionally required PKCδ activation. Although those experiments involved short‐term modulation of Wnt signalling, their findings, together with our observations, suggest that the duration and timing of Wnt pathway modulation may influence distinct aspects of the RPE phenotype. In our system, CHIR99021 treatment during prolonged iRPE culture was associated with reduced expression of transcriptional programs related to visual cycle activity, ciliogenesis, phagocytosis and other RPE‐associated signature genes, while promoting or preserving pigmentation and epithelial‐associated characteristics. These differential effects suggest that distinct aspects of RPE phenotype may require dynamic and stage‐specific regulation of Wnt signalling. It is also possible that prolonged CHIR99021 exposure influences additional downstream pathways that contribute to the epithelial‐associated characteristics observed in our cultures.

This functional divergence highlights an important limitation of using pigmentation alone as a surrogate marker of RPE maturity. Although pigmentation is considered as an RPE differentiation status [47], our findings indicate that increased pigmentation does not necessarily parallel the acquisition of all mature RPE functions. CHIR99021‐treated cells exhibited enhanced pigmentation together with several features consistent with RPE phenotype; however, selected specialized RPE‐associated programs were reduced. This observation is broadly consistent with recent single‐cell transcriptomic analyses showing that pigmentation intensity is only weakly associated with broader transcriptional programs linked to functional RPE maturity in spontaneously pigmented iRPE populations arising in culture [48]. In that study, pigmentation was proposed to represent a dynamic cellular state rather than a definitive indicator of mature RPE identity. Notably, several genes reported to positively correlate with pigmentation in spontaneously pigmented iRPE populations, including SERPINF1, C1R, CTSD, MFRP, RARRES2, TMEM109, and PSAP, were downregulated in CHIR99021‐treated cells despite their increased pigmentation, while only a limited subset showed modest or inconsistent upregulation (Figure S16). These findings suggest that the transcriptional programs associated with CHIR99021‐induced pigmentation differ from those underlying spontaneous pigmentation in iRPE cultures. Together, our results indicate that increased pigmentation may be achieved through distinct molecular mechanisms and should not be considered a standalone indicator of complete functional RPE maturation.

This study has several limitations. Many of the functional changes described here were inferred from transcriptomic analyses, and additional functional assays will be necessary to directly evaluate processes such as phagocytosis, epithelial transport, barrier integrity and visual cycle activity. In this study, transcriptomic analyses were performed using iRPE cells derived from a single iPSC line, and future studies using multiple independent donor lines will be important to establish the generalizability of these findings. In addition, because CHIR99021 inhibits GSK3, the effects observed during prolonged treatment may involve downstream signalling pathways or other GSK3‐regulated processes in addition to Wnt/β‐catenin signalling; therefore, the contribution of individual signalling pathways cannot be resolved using this pharmacological approach alone.

Despite these limitations, our findings provide insight into the effects of CHIR99021 treatment on the long‐term characteristics of iRPE cells. We also describe a simple and accessible approach for semi‐quantitative assessment of RPE pigmentation using standard bright‐field imaging, which may facilitate future studies investigating the relationship between pigmentation and cellular maturation. CHIR99021 treatment during prolonged iRPE culture promoted or preserved pigmentation, and was associated with epithelial‐related characteristics, but did not uniformly support transcriptional programs associated with specialized RPE functions. These findings indicate that increased pigmentation does not necessarily reflect broader RPE maturation and highlight the importance of stage‐specific signalling control and integrated morphological, molecular, and functional assessment when evaluating RPE maturation and optimizing long‐term culture conditions.

## Author Contributions

**Gopika S. Kumar:** Conceptualization, Data curation, Formal analysis, Methodology, Resources, Writing – original draft, Writing – review and editing; **Srijan Jayaraman:** Data curation, Formal analysis, Methodology, Writing – review and editing; **Anwar A. Palakkan:** Conceptualization, Data curation, Formal analysis, Funding acquisition, Investigation, Methodology, Project administration, Resources, Supervision, Visualization, Writing – original draft, Writing – review and editing.

## Conflicts of Interest

The authors declare no conflicts of interest.

## Supporting information


Supporting File 1

Supporting File 2

Supporting File 3

Supporting File 4

## Data Availability

The RNA sequencing datasets generated in this study have been deposited in the National Center for Biotechnology Information (NCBI) Sequence Read Archive (SRA) under BioProject accession number PRJNA1469774 and are publicly available at: https://www.ncbi.nlm.nih.gov/bioproject/PRJNA1469774.
