# Supplementary material for: CHIR99021 Treatment Promotes Pigmentation but Differentially Affects RPE‐Associated Transcriptional Programs in Human iPSC‐Derived RPE
Source: Cell Biochem Funct. 2026 Nov 15;44(11):e70343. doi: 10.1002/cbf.70343 (PMC13770262; doi:10.1002/cbf.70343)
Supplement: Supplementary file 1 [file CBF-44-e70343-s001.pdf]

CHIR99021 Treatment Promotes Pigmentation but Differentially Affects RPE-Associated Transcriptional Programs in Human iPSC-Derived RPE

Gopika S. Kumar[1], Srijan Jayaraman[1], Anwar A. Palakkan[1,2]*

[1]Department of Immunology and Stem Cell Biology, Aravind Medical Research Foundation, Anna Nagar, Madurai, Tamil Nadu, India

[2]Abate Eye Hospital, Perinthalmanna, Malappuram, Kerala, India

*Correspondence: bioanwar@gmail.com, anwar.azad@aravind.org

Supplementary File 2: Additional Data

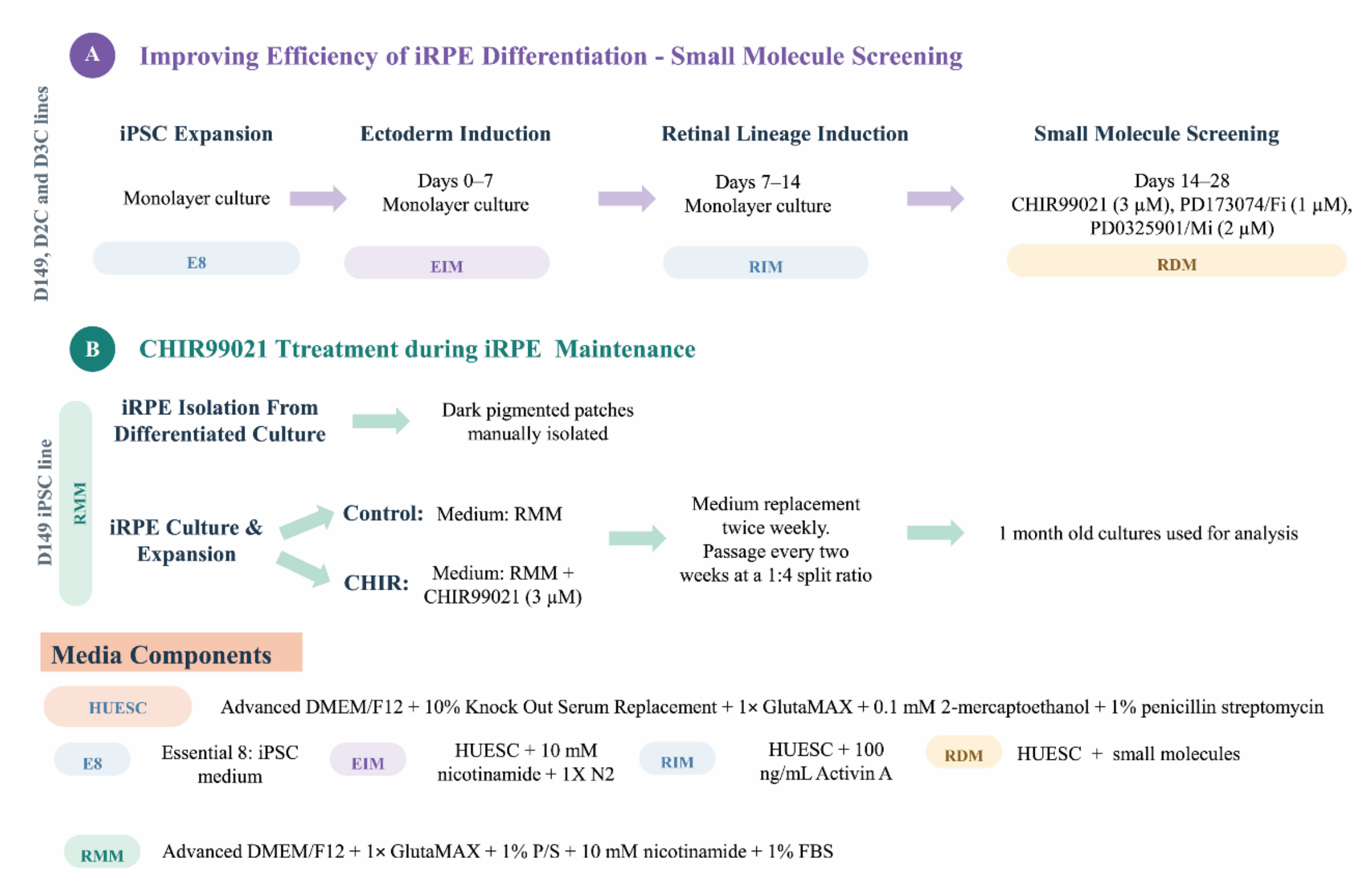

Figure S1. Schematic representation of the experimental workflow used for iRPE differentiation and subsequent CHIR99021 treatment. iPSCs were differentiated into iRPE cells using the established differentiation protocol, followed by small-molecule treatment during the final 14 days of differentiation. CHIR99021, PD173074, and PD0325901 were evaluated individually and in combination to identify the treatment condition associated with enhanced generation of pigment-positive iRPE cells. Based on the initial screening results, CHIR99021 was selected for subsequent experiments. For the prolonged-culture experiments, CHIR99021 treatment was continued during the maturation/maintenance phase and through subsequent serial passaging to assess the effects of CHIR99021 on pigmentation, cellular characteristics, and transcriptional programs.

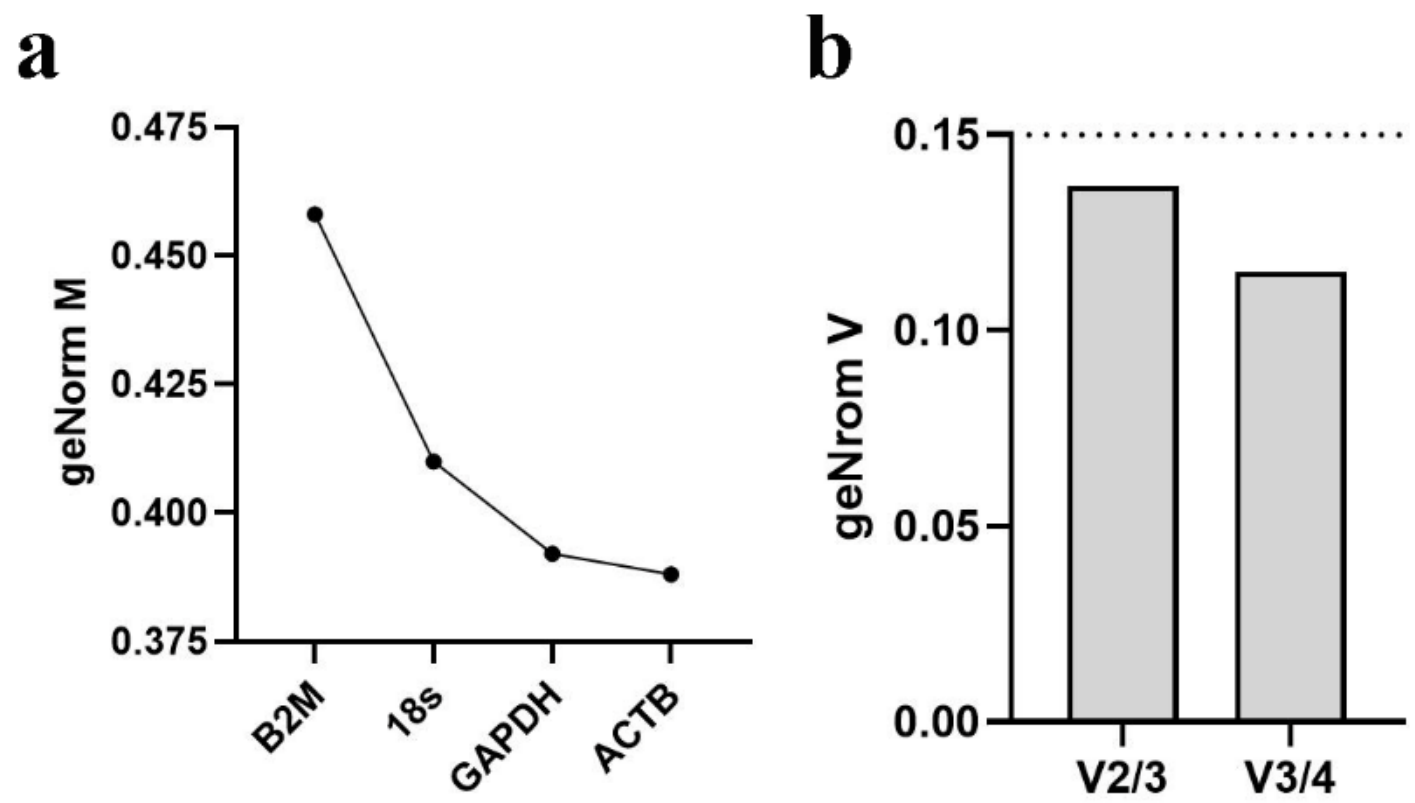

Figure S2. geNorm analysis of reference gene stability. (a) Graph showing the average expression stability (M value) of the remaining reference genes. The stability ranking of the reference genes was: ACTB > GAPDH > 18s > B2M. (b) Graph showing the optimal number of reference genes for normalization. The optimal number was determined to be two (geNorm V < 0.15 when comparing normalization factors based on the two or three most stable genes). For subsequent qPCR analyses, the geometric mean of ACTB and GAPDH was used as the normalization factor.

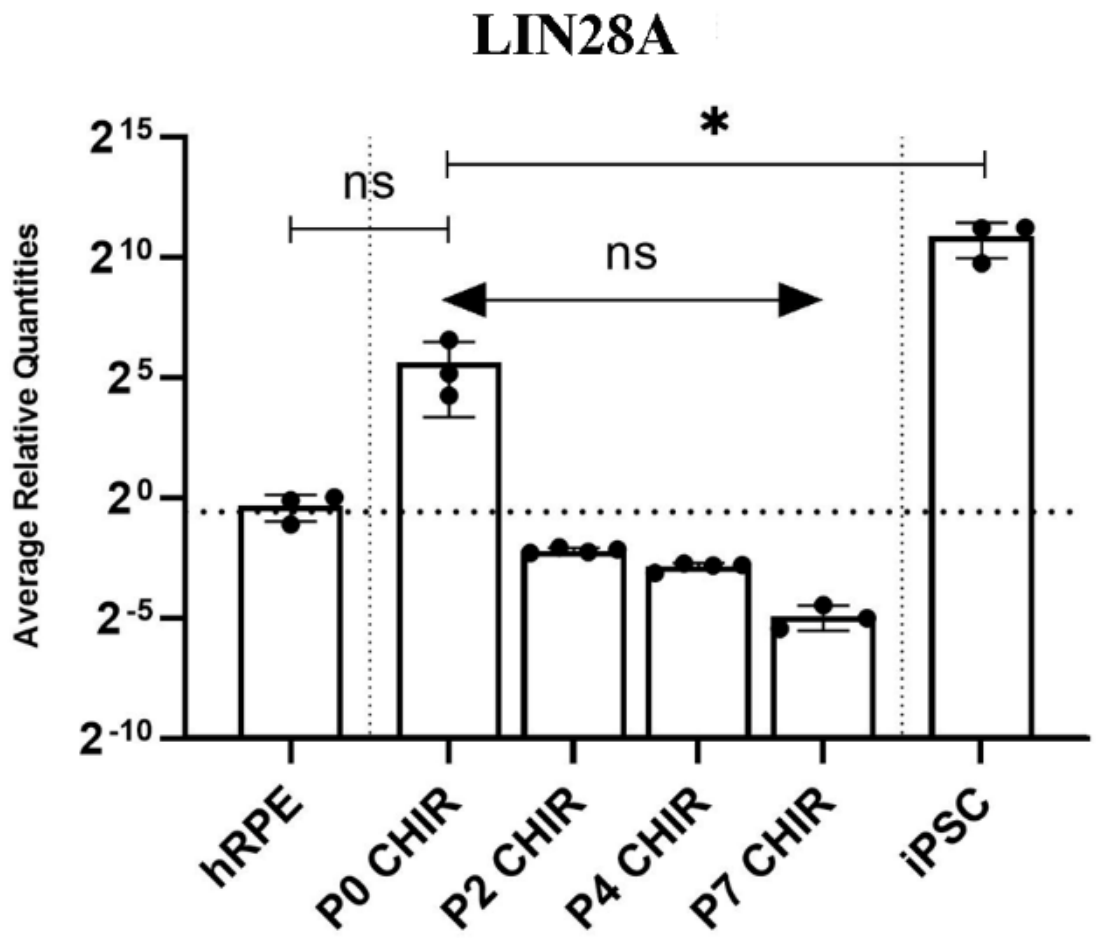

Figure S3: *LIN28A* expression in iPSCs and RPE cultures. *LIN28A* expression was highest in iPSCs and minimal in primary human RPE (hRPE). iRPE at passage 0 (P0) showed higher *LIN28A* expression than hRPE, whereas CHIR99021 treatment (CHIR) resulted in reduced *LIN28A* expression in iRPE cultures at P2, P4 and P7. Quantitative PCR data are presented as normalized relative quantity (RQ) calculated relative to hRPE using Qbase+ software. At least three independent biological replicates were analysed per condition, and data are shown as mean ± standard deviation. Statistical significance was determined by one-way ANOVA with Tukey's multiple-comparisons correction ($p < 0.0001$). * indicates statistically significant differences, while non-significant comparisons are denoted as "ns".

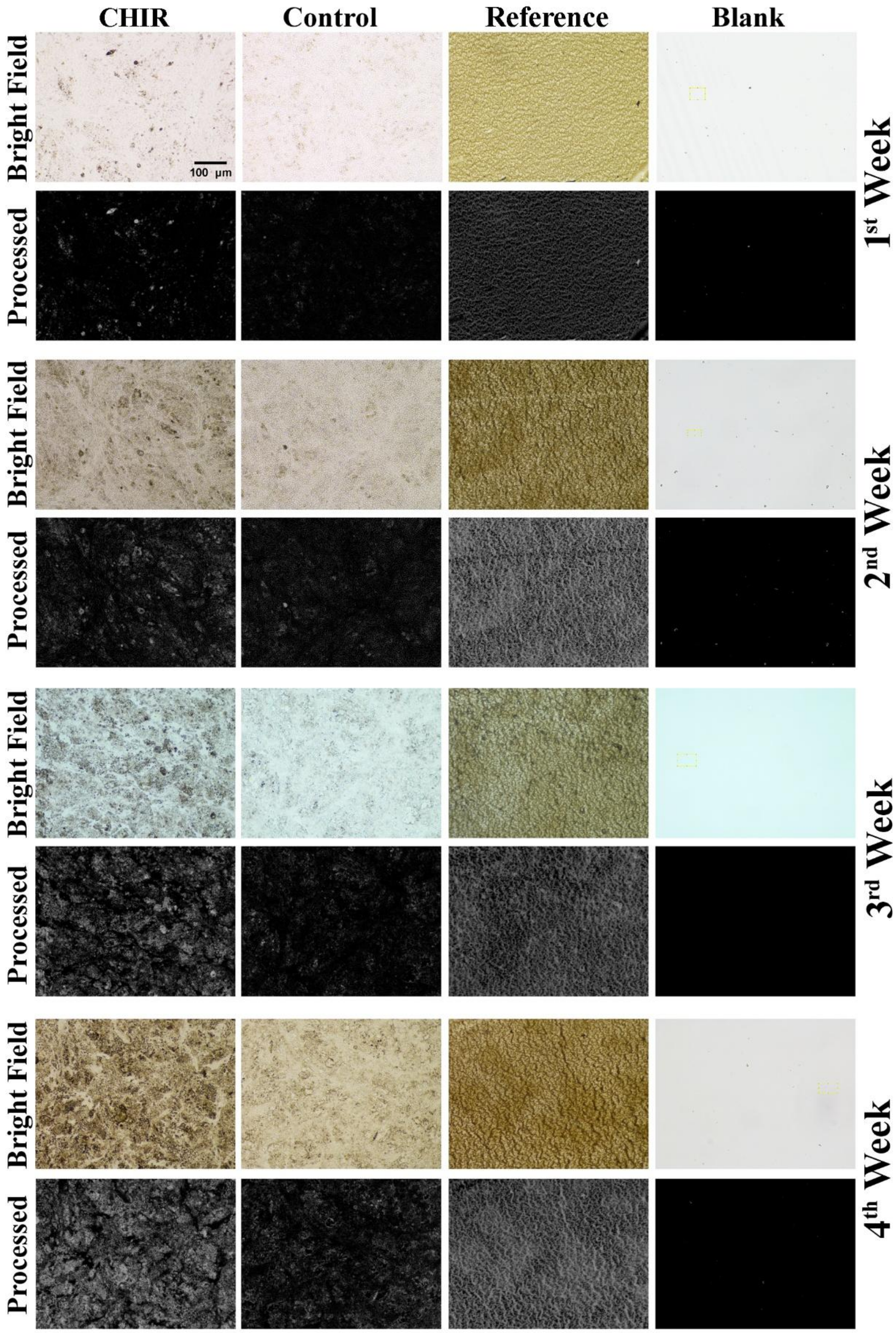

Figure S4. Image-based assessment of relative optical pigmentation in iRPE cells during culture. Representative bright-field images and their corresponding processed images, generated using an ImageJ analysis pipeline (background subtraction, grayscale conversion, and image inversion) by running the script provided in the supplementary file, are shown for blank, melanin reference, control, and CHIR99021 (CHIR)-treated iRPE cultures at weeks 1–4 of culture. Relative optical pigmentation was normalized to the melanin reference and expressed as percentage of reference value. The resulting values were used for the semi-quantitative analysis and for plotting the graph shown in Figure 2a.

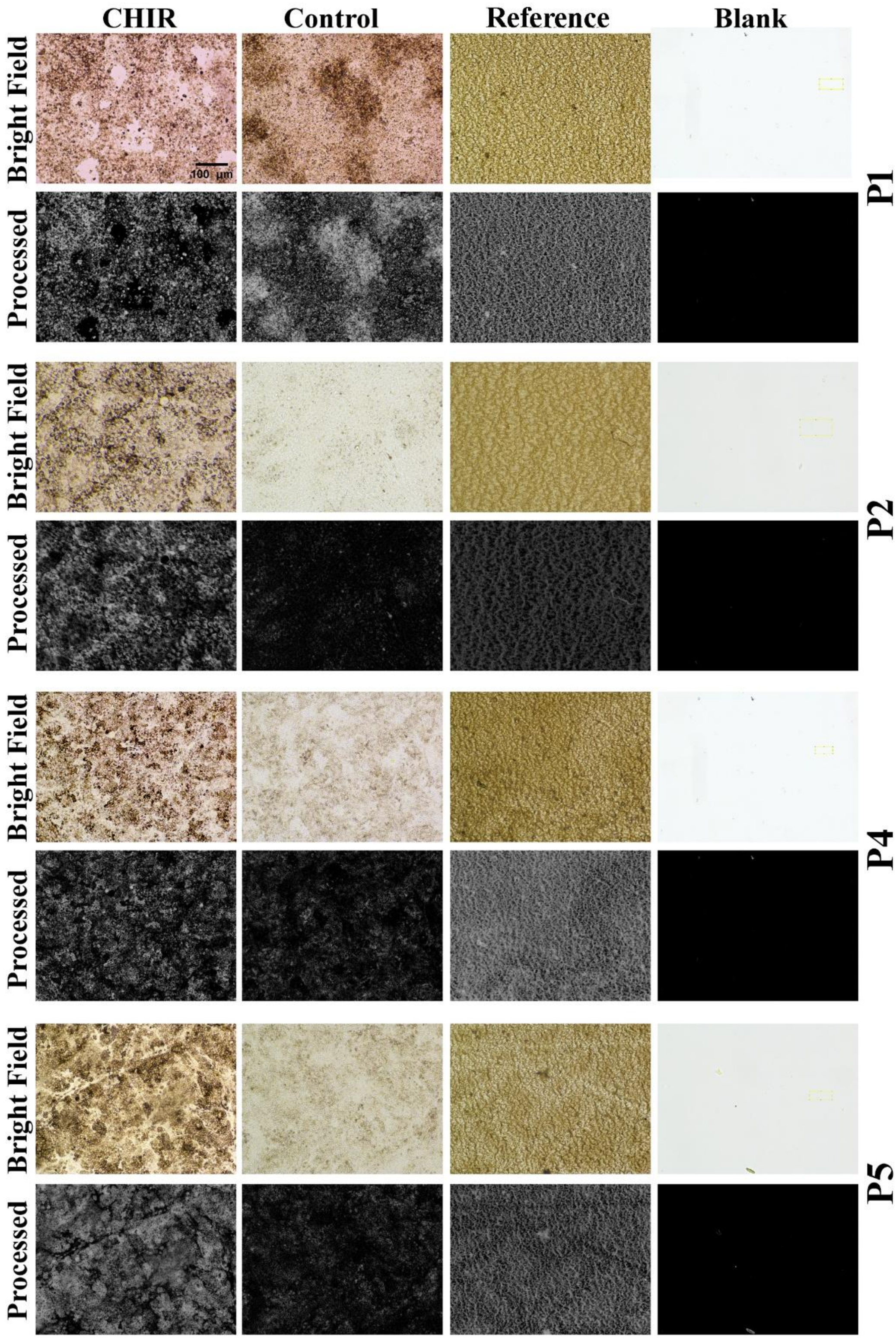

Figure S5. Image-based assessment of relative optical pigmentation in iRPE cells at different passages. Representative bright-field images and their corresponding processed images, generated using an ImageJ analysis pipeline (background subtraction, grayscale conversion, and image inversion) by running the script provided in the supplementary algorithm, are shown for blank, melanin reference, control, and CHIR99021 (CHIR)-treated iRPE cultures at different passages (P1, P2, P4 and P5). Relative optical pigmentation was normalized to the melanin reference and expressed as a percentage of the reference value. The resulting values were used for semi-quantitative analysis and for plotting the graph shown in Figure 2b.

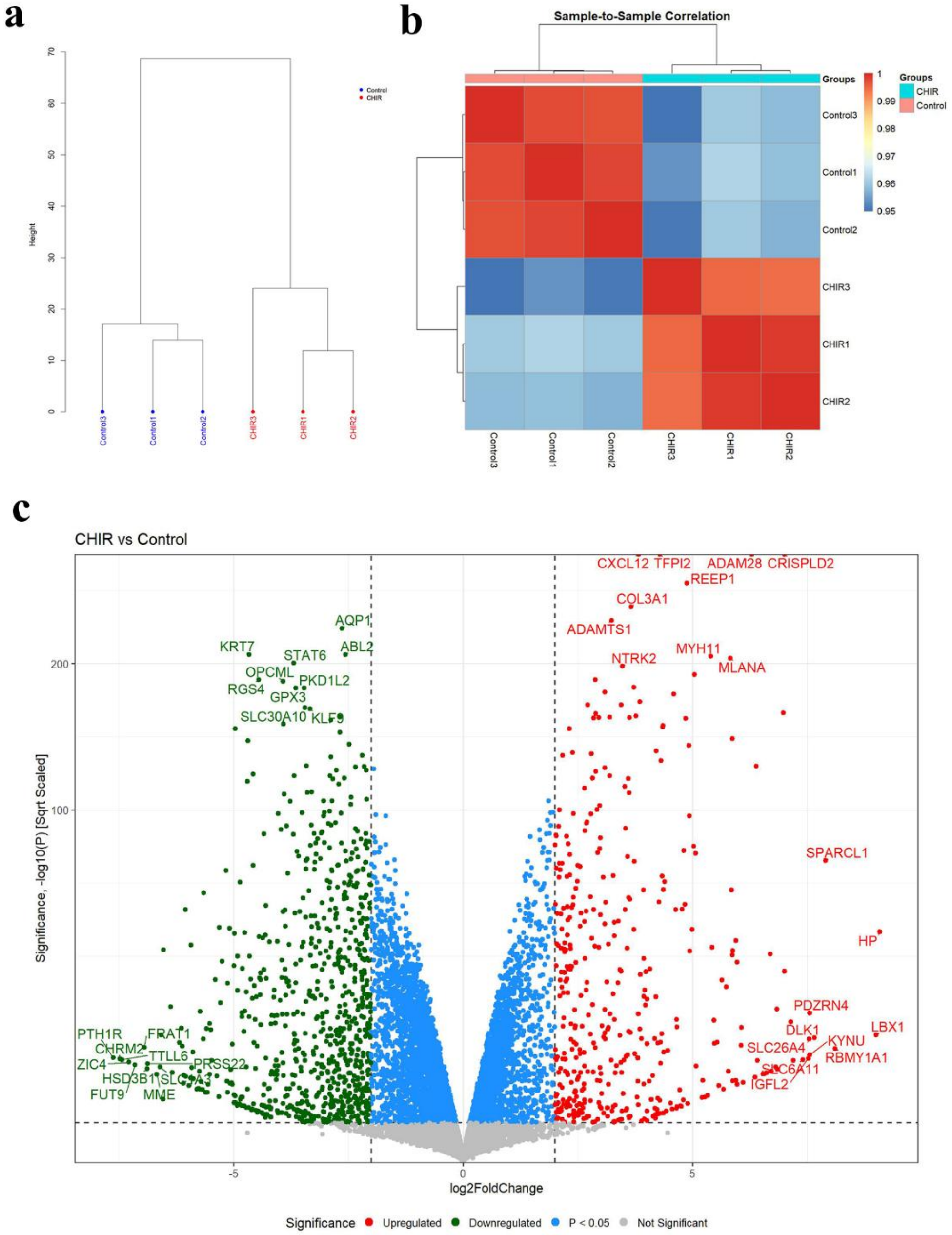

Figure S6. CHIR99021 treatment induces distinct transcriptional changes in iRPE cells.
(a) Unsupervised hierarchical clustering and dendrogram analysis of RNA-seq data from passage 4 iRPE cells continuously treated with CHIR99021 (CHIR) from differentiation through passage 4 and passage-matched control iRPE cells (Control). Samples cluster according to treatment condition, with biological replicates clustering closely within each group. (b) Sample-to-sample correlation heatmap showing high correlations among biological replicates within each condition and lower correlation between CHIR-treated and control iRPE samples. (c) Volcano plot depicting differential gene expression between CHIR99021-treated (CHIR) and control iRPE cells. Grey, blue, red, and green points indicate non-significant genes ($p \geq 0.05$), significant genes with $|log2FC| < 2$, significantly upregulated genes ($p < 0.05$, $log2FC > 2$), and significantly downregulated genes ($p < 0.05$, $log2FC < -2$), respectively. The y-axis was square-root transformed for improved visualization. Labels represent the top 10 genes by positive/negative log2FC and the top 10 by statistical significance for each direction, yielding up to 40 labelled genes.

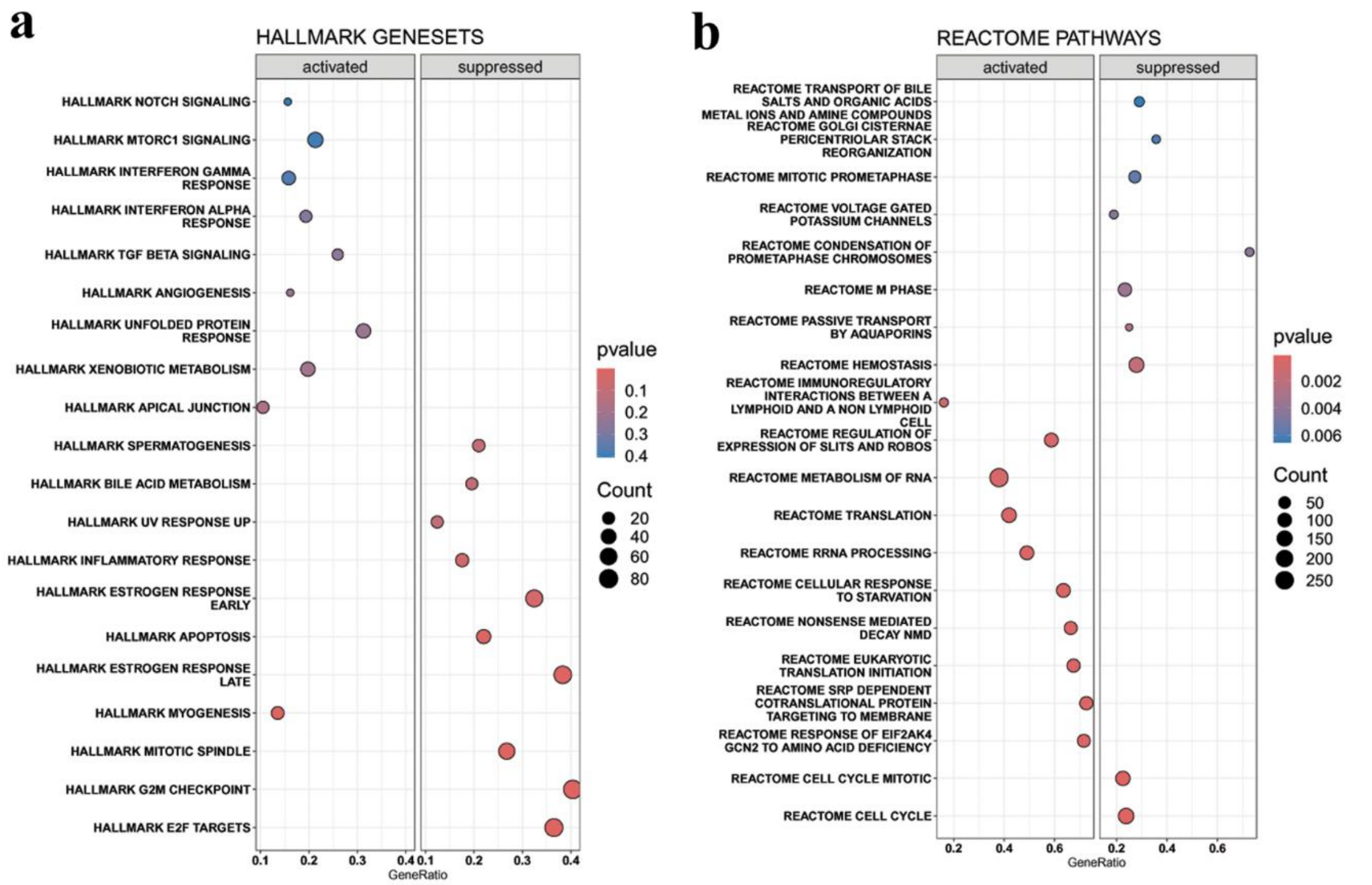

Figure S7. Hallmark and Reactome pathway enrichment analysis following CHIR99021 treatment. Bubble plots depict significantly enriched (A) Hallmark gene sets and (B) Reactome pathways derived from differentially expressed genes following prolonged CHIR99021treatment (FDR < 0.05). A total of 417 upregulated genes ($\log_2$ fold change ≥ 2) and 794 downregulated genes ($\log_2$ fold change ≤ -2) were included in the analysis. Enriched and reduced gene sets/pathways are indicated. Bubble size represents the number of genes associated with each pathway, while colour denotes statistical significance (p-value).

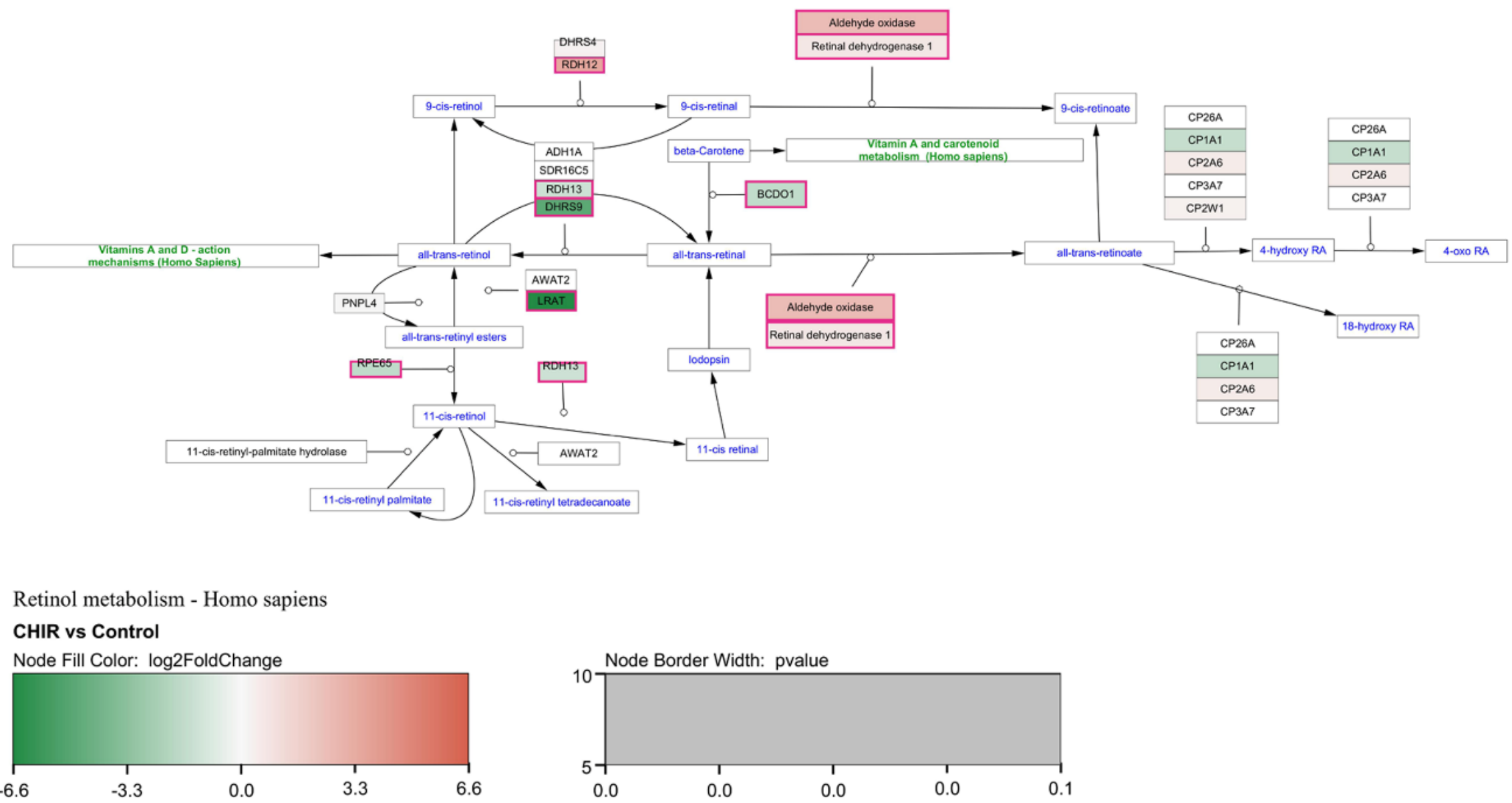

Figure S8. Pathway-based visualization of gene expression changes mapped onto the retinoid metabolism/visual cycle pathway. Differential expression between CHIR99021-treated (CHIR) and control conditions is represented by colour intensity, where red indicates upregulation and green indicate downregulation relative to control. Several components of the retinal visual cycle show altered expression, indicating that prolonged CHIR99021 treatment influences visual cycle–associated pathways.

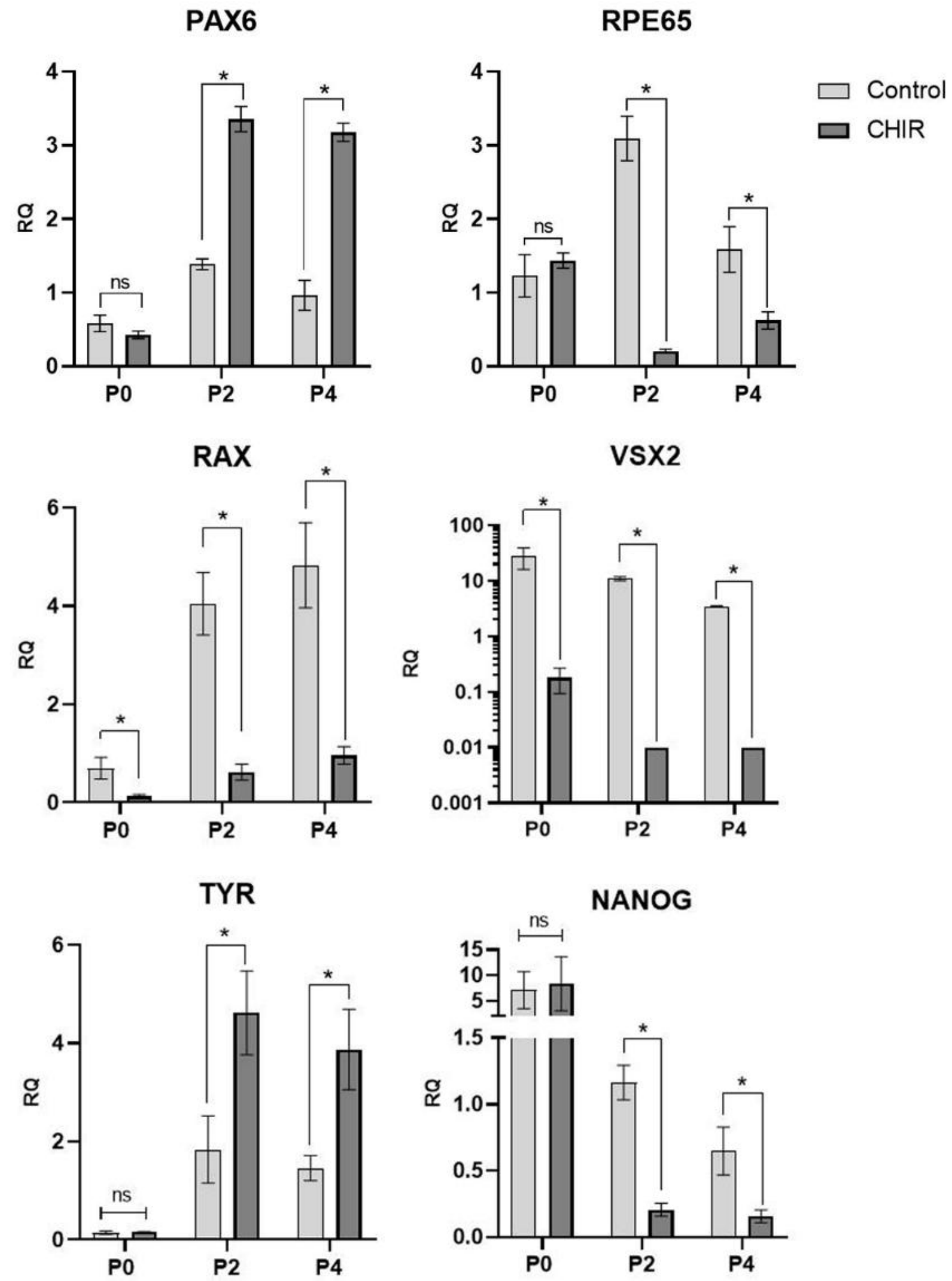

Figure S9. qPCR analysis of CHIR99021 treated iRPE cells compared to control at different passages. Quantitative PCR analysis showing relative quantity (RQ) values of *PAX6, RPE65, RAX, VSX2, TYR,* and *NANOG* at different passages—differentiation culture (P0), Passage 2 (P2), and Passage 4 (P4)—under CHIR99021-treated (CHIR) and control conditions. Data are presented as mean ± SD (n = 3). Statistical significance was assessed using one-way ANOVA. * indicates statistically significant differences (P ≤ 0.05), while non-significant comparisons are denoted as “ns”.

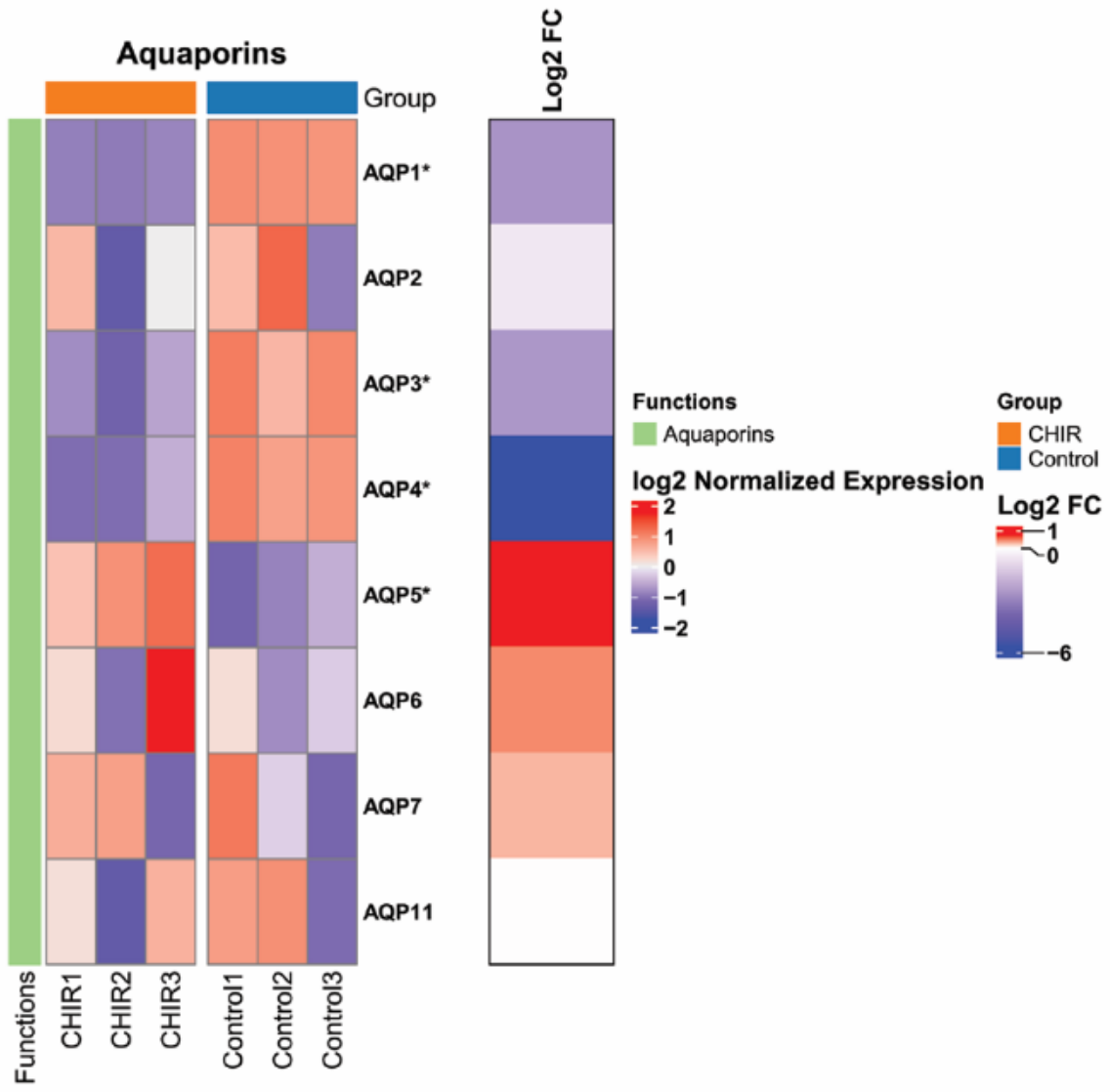

Figure S10. Expression profile of aquaporin (AQP) genes in CHIR99021-treated and control iRPE cells. Heatmap showing the log_2 normalized expression of aquaporin family genes in iRPE cells cultured under control conditions (Control1–Control3) or treated with CHIR99021 (CHIR1–CHIR3). Each column represents a biological replicate, and each row represents an aquaporin gene. Colour intensity reflects relative gene expression levels following normalization (red: higher expression; blue: lower expression). The panel on the right shows the corresponding log_2 fold change (Log_2FC) between CHIR-treated and control samples. Genes analysed include AQP1, AQP2, AQP3, AQP4, AQP5, AQP6, AQP7, and AQP11, which are associated with water transport and fluid homeostasis in RPE cells. Most aquaporins showed reduced expression in CHIR-treated cells relative to controls, whereas AQP5 exhibited increased expression. Heatmaps depict normalized expression levels, with adjacent bars showing log fold change (Log FC). Asterisks (*) indicate significantly altered genes.

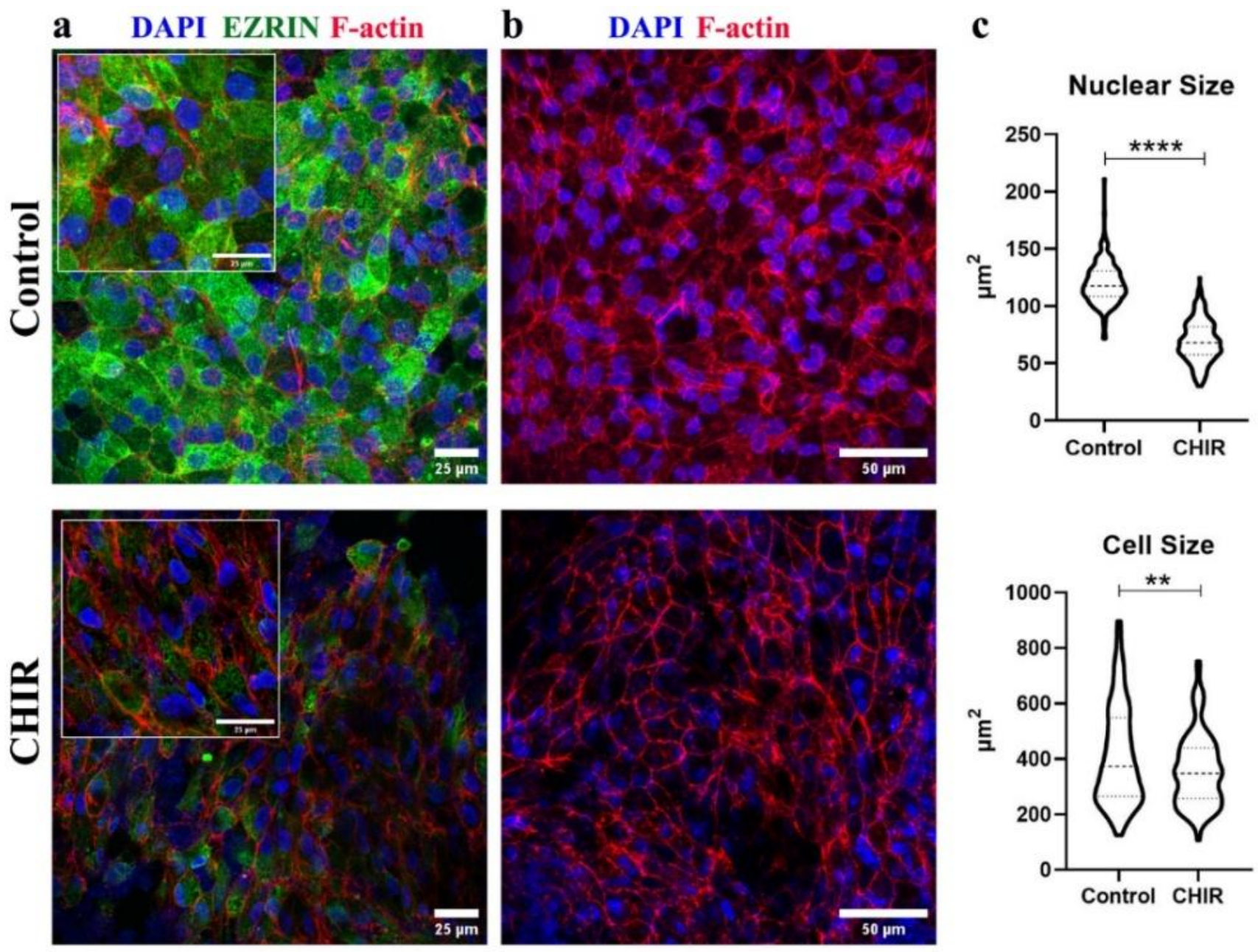

Figure S11. CHIR99021 treatment induces morphological changes in iRPE cells.
Representative immunofluorescence images showing changes in (a) primary cilium (EZRIN) and (b) F-actin localization in iRPE cells following CHIR99021-treatment (CHIR) compared to control. Insets show higher-magnification views. CHIR treatment is associated with reduced EZRIN expression (a) and increased localization of F-actin at cell borders (b). Scale bar: 10 µm. (c) Image analysis of nuclear and cellular size based on DAPI and F-actin staining shows that CHIR treatment decreases nuclear and cellular size. Statistical significance was assessed using one-way ANOVA (n = 250 cells). **** indicates $P < 0.0001$, and ** indicates $P < 0.01$.

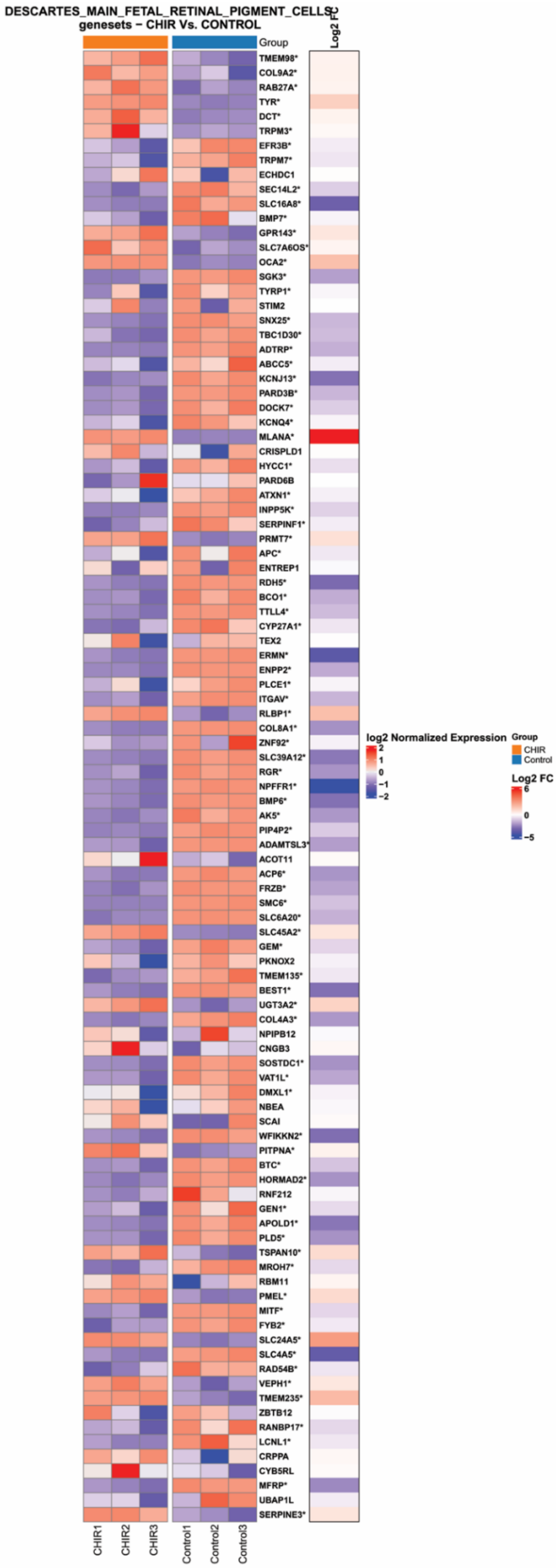

Figure S12. Expression of genes from the DESCARTES MAIN FETAL RETINAL PIGMENT CELLS gene set in CHIR99021-treated and control iRPE cells. Heatmap showing normalized expression of genes

from the DESCARTES_MAIN_FETAL_RETINAL_PIGMENT_CELLS gene set detected in both CHIR99021-treated and control iRPE cells based on a raw count threshold of >20. Three biological replicates were analysed for each condition. Of the 265 genes in the gene set, 104 were detected in CHIR99021-treated cells and 105 in control cells, with 101 genes detected in both conditions. The heatmap illustrates normalized expression across individual biological replicates, with adjacent bars showing log fold change (Log FC). Asterisks (*) indicate significantly altered genes.

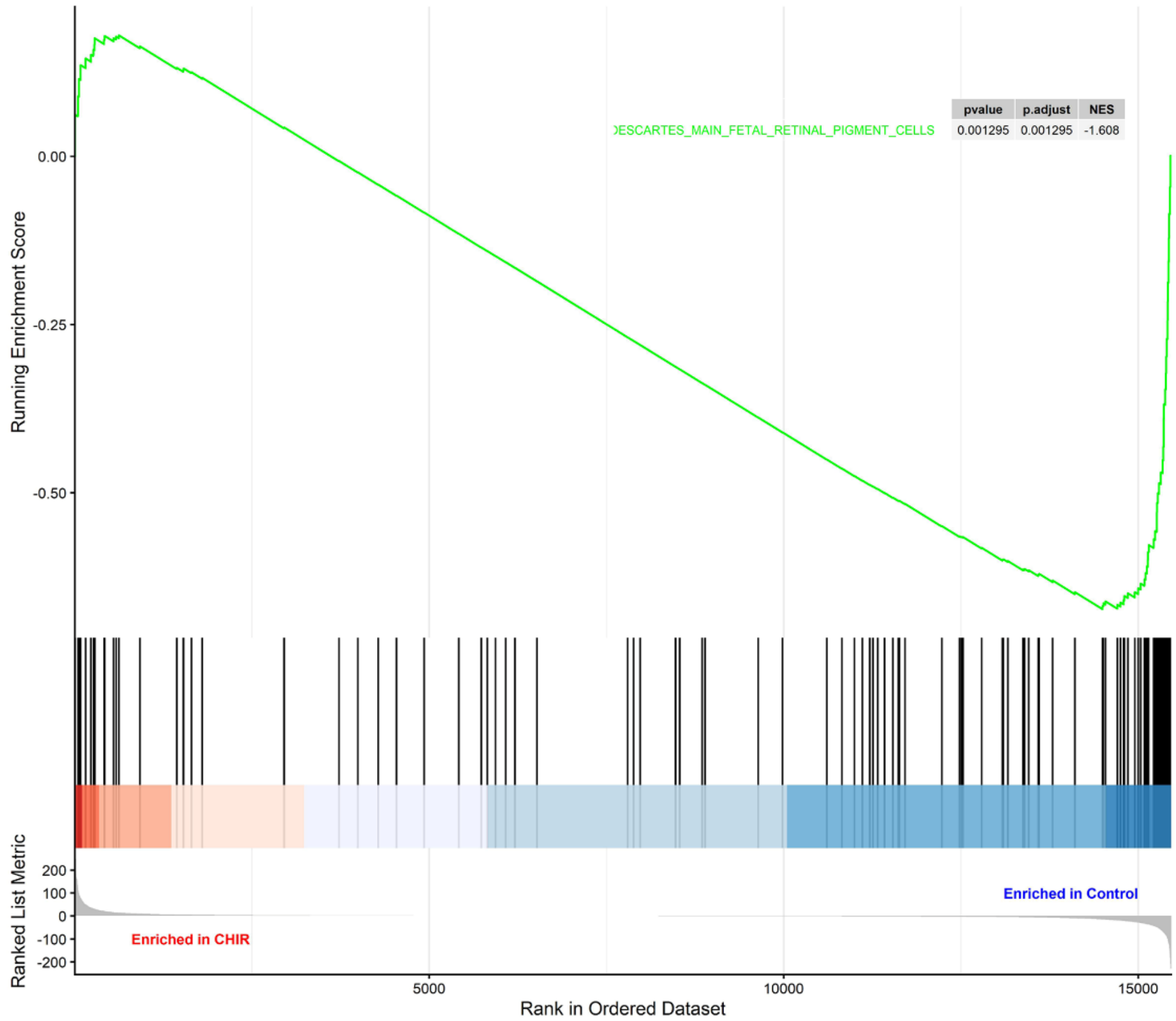

Figure S13. GSEA of the DESCARTES_MAIN_FETAL_RETINAL_PIGMENT_CELLS gene set in CHIR99021-treated and control iRPE cells. Enrichment plots show the distribution of genes from the DESCARTES_MAIN_FETAL_RETINAL_PIGMENT_CELLS gene set within the ranked list of genes according to differential expression between CHIR99021-treated and control iRPE cells. Negative normalized enrichment scores (NES) indicate enrichment toward the control group. The ranked-list metric (bottom panel) represents the differential-expression ranking between the two conditions, with genes preferentially enriched in CHIR99021-treated cells shown on the left (red) and genes preferentially enriched in passage-matched control cells shown on the right (blue).

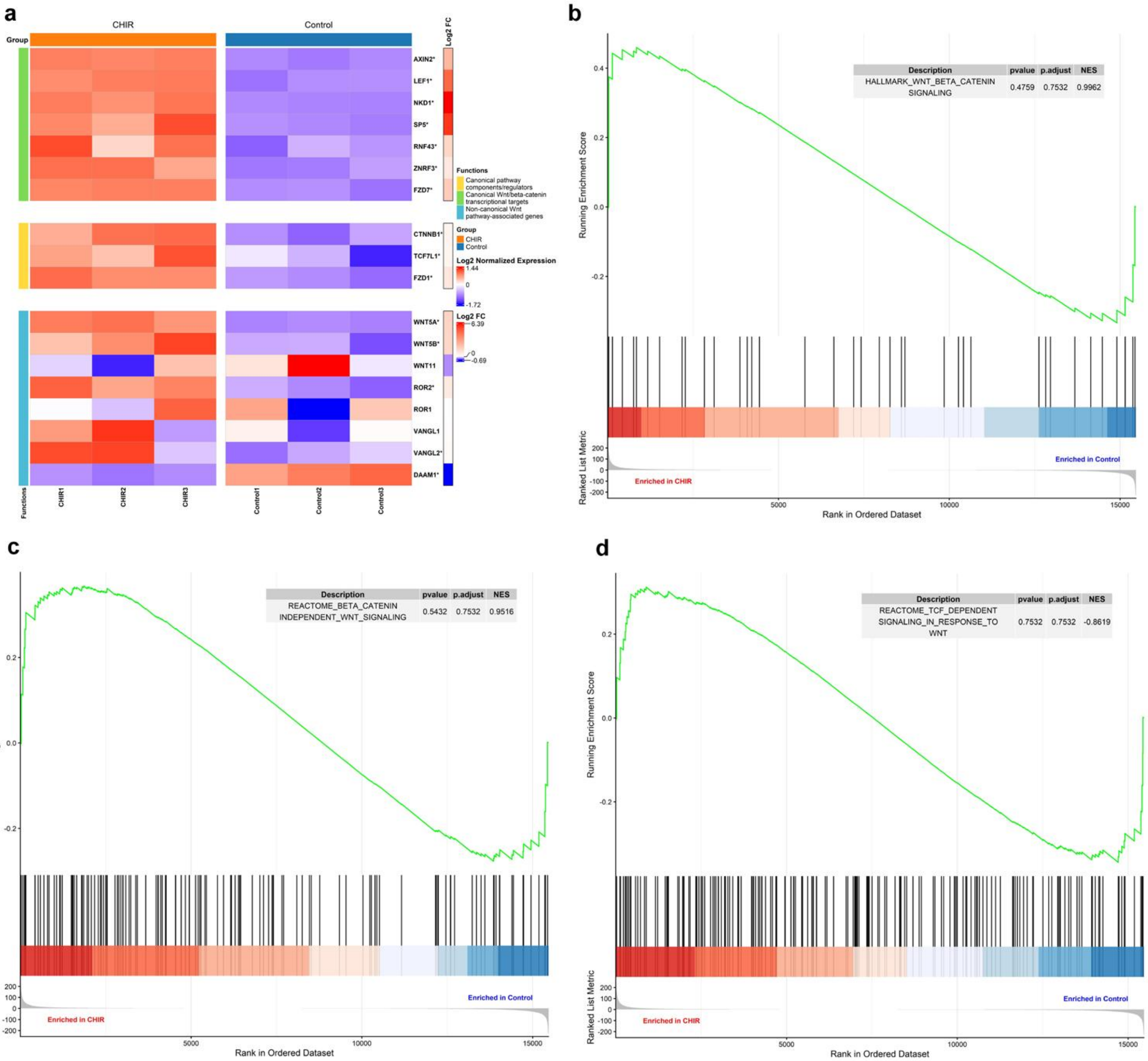

Figure S14. Transcriptional remodelling of Wnt-associated genes following CHIR99021 treatment. (a) Heatmap showing the expression of selected Wnt-associated genes in CHIR99021-treated (CHIR) and control iRPE cells. RNA-seq data from three biological replicates of CHIR99021-treated cells (CHIR1–CHIR3) and control cells (Control1–Control3) are shown. Colour intensity represents relative gene expression following normalization (red, higher expression; blue, lower expression). The panel on the right indicates the corresponding $\log_2$ fold change ($\log_2$FC) between CHIR99021-treated and control conditions. Significantly changed genes are indicated by an asterisk (*). (b–d) Gene Set Enrichment Analysis (GSEA) of Wnt-associated gene sets comparing CHIR99021-treated and control iRPE cells, showing the Hallmark Wnt/β-catenin signalling gene set (b), Reactome β-catenin-independent Wnt signalling gene set (c), and Reactome TCF-dependent signalling in response to Wnt gene set (d). Enrichment plots depict genes ranked according to differential expression between CHIR99021-treated and control groups. Positive and negative normalized enrichment scores (NES) indicate enrichment toward CHIR99021-treated and control cells, respectively. The ranked list metric (bottom panel) reflects differential gene expression between the two conditions, with genes preferentially enriched in CHIR99021-treated cells shown on the left (red) and genes preferentially enriched in passage-matched control cells shown on the right (blue).

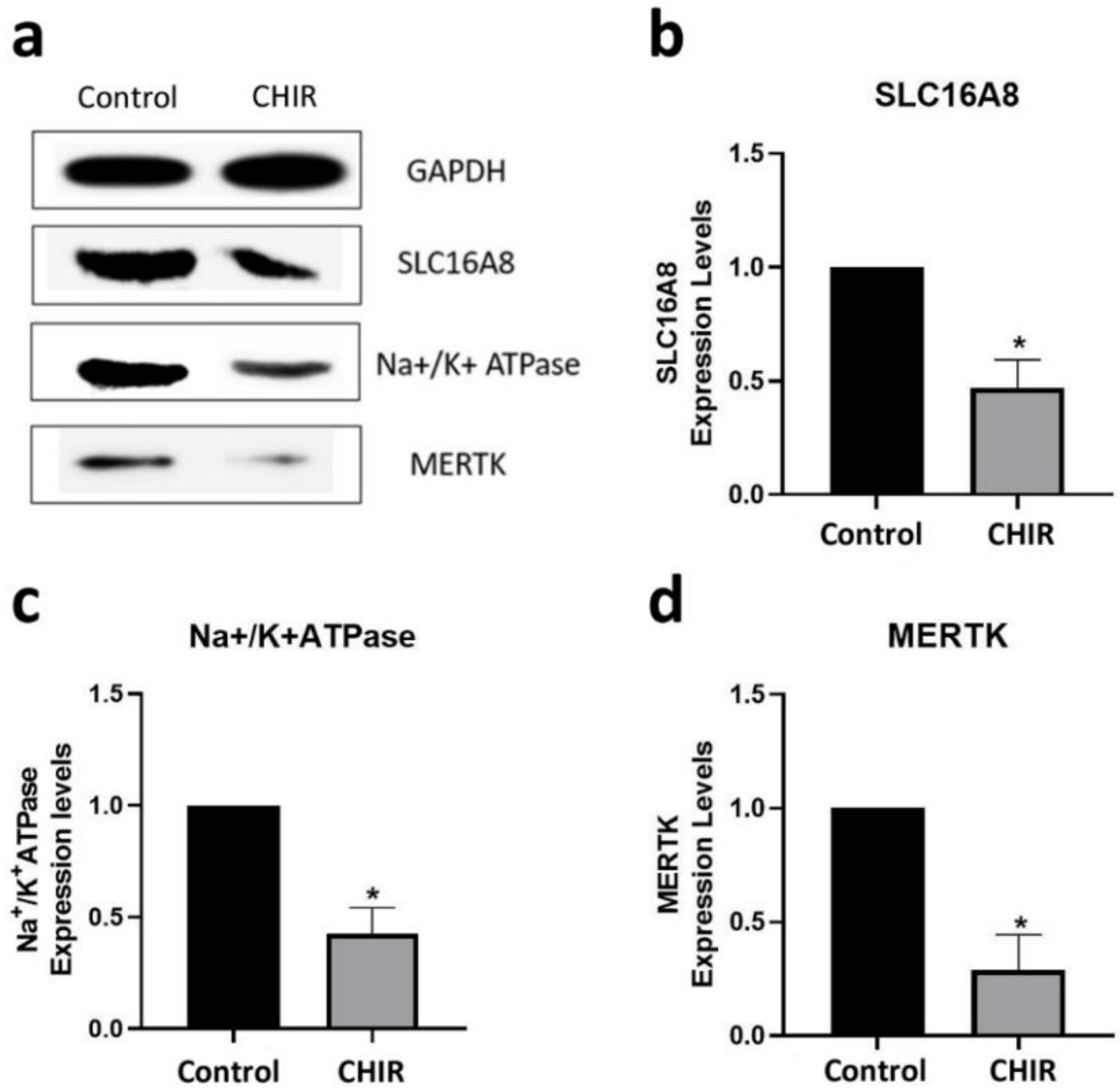

Figure S15. CHIR99021 treatment alters expression of selected RPE proteins in iRPE cells. (a) Representative western blot images showing expression of the RPE-associated proteins SLC16A8 (MCT3), Na^+/K^+-ATPase, and MERTK in control and CHIR99021-treated iRPE cells. GAPDH was used as the loading control. Sustained CHIR99021 treatment resulted in reduced expression of these proteins compared with untreated control cells. (b–d) ImageJ-based densitometric quantification of SLC16A8, Na^+/K^+-ATPase, and MERTK protein expression normalized to GAPDH and expressed relative to control cells. Data are presented as mean ± SD. Statistical significance was assessed using an unpaired t-test (n = 3 for SLC16A8; n = 2 for Na^+/K^+-ATPase and MERTK). *P < 0.05 versus control.

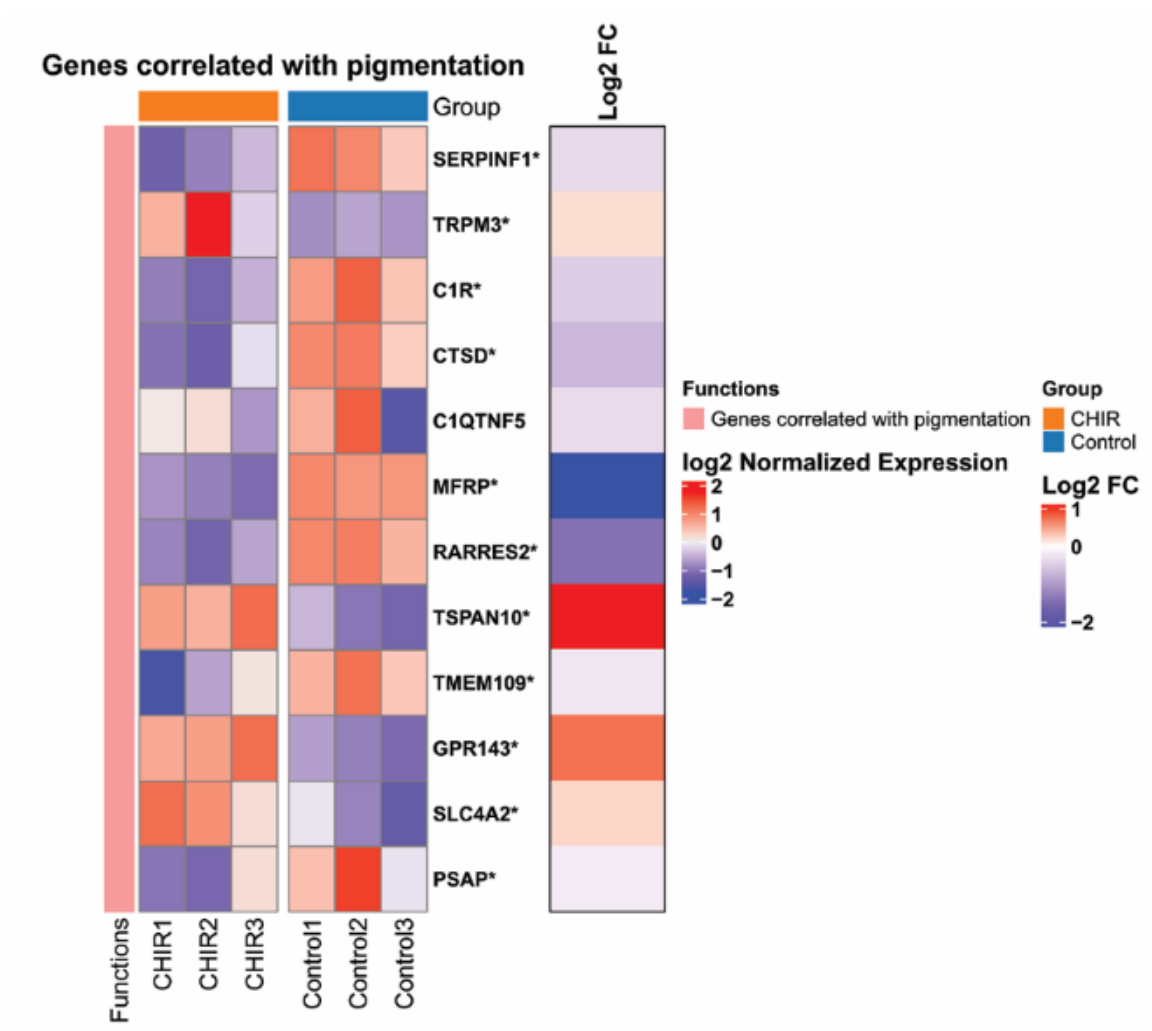

Figure S16. Expression level of genes previously reported to correlate with RPE pigmentation. Heatmap showing the $\log_2$ normalized expression levels of selected genes previously reported to correlate with RPE pigmentation. RNA-seq data from three biological replicates of CHIR-treated cells (CHIR1–CHIR3) and control cells (Control1–Control3) are shown. Colour intensity represents relative gene expression following normalization (red: higher expression; blue: lower expression). The panel on the right indicates the corresponding $\log_2$ fold change (Log_2FC) between CHIR-treated and control conditions. Significantly changed genes are indicated by an asterisk (*).
