# Supplementary material for: CHIR99021 Treatment Promotes Pigmentation but Differentially Affects RPE‐Associated Transcriptional Programs in Human iPSC‐Derived RPE
Source: Cell Biochem Funct. 2026 Nov 15;44(11):e70343. doi: 10.1002/cbf.70343 (PMC13770262; doi:10.1002/cbf.70343)
Supplement: Supplementary file 2 [file CBF-44-e70343-s003.pdf]

CHIR99021 Treatment Promotes Pigmentation but Differentially Affects RPE-Associated Transcriptional Programs in Human iPSC-Derived RPE

Gopika S. Kumar[1], Srijan Jayaraman[1], Anwar A. Palakkan[1,2*]

[1]Department of Immunology and Stem Cell Biology, Aravind Medical Research Foundation, Anna Nagar, Madurai, Tamil Nadu, India
[2]Abate Eye Hospital, Perinthalmanna, Malappuram, Kerala, India

[*]Correspondence: bioanwar@gmail.com, anwar.azad@aravind.org

Supplementary File 1: <u>ImageJ macro for quantitative pigmentation analysis</u>

This ImageJ macro performs semi-automated quantitative analysis of pigmentation in retinal pigment epithelial (RPE) cell images. Bright-field images of iRPE cells, along with blank and melanin standard images in TIFF format, are required for processing. The macro automatically identifies blank and standard images based on file naming (e.g., *blank 1.tif*, *blank 2.tif* for blank replicates and *standard 1.tif*, *standard 2.tif* for standard replicates). For each blank image, the user manually defines a background region of interest (ROI), which is used to normalize image brightness; the blank intensity is considered as background and subtracted from all images. Images are then converted to 8-bit grayscale, inverted, and processed to obtain mean intensity, area, and integrated density (IntDen). Standard images are first analyzed to calculate an average reference IntDen, which is subsequently used to compute percentage pigmentation for experimental samples. The macro saves ROI-annotated images, processed signal images, and exports all quantitative measurements to a CSV file for downstream analysis

```
// Functions
// Function to save the image with ROI
function saveImageWithROI(Title, saveDir) {
    savePath = saveDir + Title;
    saveAs("tiff", savePath);
    if (File.exists(savePath)) {
        print("Saved ROI image: " + savePath);
    } else {
        print("Error saving ROI image: " + savePath);
    }
}

// Function to save the processed image
function saveImage(Title, saveDir) {
    savePath = saveDir + Title;
    saveAs("tiff", savePath);
    if (File.exists(savePath)) {
        print("Saved Signal image: " + savePath);
    } else {
```

```
        print("Error saving Signal image: " + savePath);
    }
    print("", "\n");
}

// Function to process individual images and return intensity-related data as an array
function processImage(imagePath) {
    open(imagePath);
    Title = getTitle();

    waitForUser("Please create a selection to indicate the background, then click OK to proceed.");
    roiManager("Add");

    roiManager("select", 0);
    Roi.setStrokeWidth(4);
    run("Flatten");

    saveImageWithROI(Title, ROIImage);

    close();
    run("Select None");
    selectImage(Title);

    run("8-bit");
    run("Invert");

    roiManager("select", 0);
    Roi.setStrokeWidth(0);
    roiManager("Measure");
    Background = getResult("Mean", 0);

    run("Select None");
    run("Measure");
    Int = getResult("Mean", 1);

```

```
    run("Subtract...", "value=" + Background);
    run("Measure");
    Sig    = getResult("Mean", 2);
    Area   = getResult("Area", 2);
    IntDen = getResult("IntDen", 2);

    saveImage(Title, SignalImage);

    return newArray(Background, Int, Sig, Area, IntDen);
}

// Main macro starts here
print("\\Clear");

inDir = getDirectory("Select the folder");
print("Input folder is " + inDir);
print("", "\n");

list   = getFileList(inDir);
length = list.length;

outDir = inDir + "processed/";
File.makeDirectory(outDir);

ROIImage    = outDir + "ROIImage/";
SignalImage = outDir + "SignalImage/";
File.makeDirectory(ROIImage);
File.makeDirectory(SignalImage);

WriteResult = outDir + "Results.csv";
f = File.open(WriteResult);
print(f, "Image,Background,Raw Intensity,Signal Mean,Area,IntDen,Percentage Pigmentation,\n");

standardIntDenSum = 0;
standardCount     = 0;
```

```
nstdcount         = 0;

for (j = 0; j < length; j++) {
    if (endsWith(list[j], ".tif") && startsWith(list[j], "standard ")) {
        imagePath = inDir + list[j];
        results = processImage(imagePath);

        Background = results[0];
        Int        = results[1];
        Sig        = results[2];
        Area       = results[3];
        IntDen     = results[4];

        close("ROI Manager");
        standardIntDenSum += IntDen;
        standardCount++;

        print(f, list[j] + "," + Background + "," + Int + "," + Sig + "," + Area + "," + IntDen + ",\n");

        close();
        close("Results");
        nstdcount++;
    }
}

standardIntDen = standardIntDenSum / standardCount;
print("Average Standard IntDen: " + standardIntDen);
print("", "\n");

for (j = 0; j < length; j++) {
    if (endsWith(list[j], ".tif") && !startsWith(list[j], "standard ")) {
        imagePath = inDir + list[j];
        results = processImage(imagePath);

```

```
        Background = results[0];
        Int        = results[1];
        Sig        = results[2];
        Area       = results[3];
        IntDen     = results[4];

        close("ROI Manager");

        if (nstdcount < 1) {
            percentagePigmentation = "Nil";
        } else {
            percentagePigmentation = (IntDen / standardIntDen) * 100;
        }

        print(f, list[j] + "," + Background + "," + Int + "," + Sig + "," + Area + "," + IntDen + "," + percentagePigmentation + "\n");

        if (nImages > 0) {
            close();
        }

        close("Results");
    }
}

File.close(f);
print("Processing complete.");
```
