# Supplementary material for: CHIR99021 Treatment Promotes Pigmentation but Differentially Affects RPE‐Associated Transcriptional Programs in Human iPSC‐Derived RPE
Source: Cell Biochem Funct. 2026 Nov 15;44(11):e70343. doi: 10.1002/cbf.70343 (PMC13770262; doi:10.1002/cbf.70343)
Supplement: Supplementary file 3 [file CBF-44-e70343-s002.docx]

|  | **Gene** | **Forward** | **Reverse** | **Tm** |
| --- | --- | --- | --- | --- |
| 1 | ACTB | CACCATTGGCAATGAGCGGTTC | AGGTCTTTGCGGATGTCCACGT | 60℃ |
| 2 | 18S | GAGGATGAGGTGGAACGTGT | TCTTCAGTCGCTCCAGGTCT | 60℃ |
| 3 | B2M | CATTCCTGAAGCTGACAGCATTC | TGCTGGATGACGTGAGTAAACC | 60℃ |
| 4 | GAPDH | GAAGGTGAAGGTCGGAGTC | GAAGATGGTGATGGGATTTC | 60℃ |
| 5 | PAX6 | GCCAGCAACACACCTAGTCA | TGTGAGGGCTGTGTCTGTTC | 60℃ |
| 6 | RPE65 | AGCACTGAGTTGAGCAAGCA | GGCCTGTCTCACAGAGGAAG | 60℃ |
| 7 | RAX | GGCAAGGTCAACCTACCAGAG | CATGGAGGACACTTCCAGCTT | 60℃ |
| 8 | VSX2 | CTGCCGGAAGACAGGATACA | TAGAGCCCATACTCCGCCA | 60℃ |
| 9 | TYR | GTGTAGCCTTCTTCCAACTCAG | GTTCCTCATTACCAAATAGCATCC | 60℃ |
| 10 | NANOG | CAAAGGCAAACAACCCACTT | TCTGCTGGAGGCTGAGGTAT | 60℃ |

Table S2: List of primer used for qPCR.
