# Supplementary material for: CHIR99021 Treatment Promotes Pigmentation but Differentially Affects RPE‐Associated Transcriptional Programs in Human iPSC‐Derived RPE
Source: Cell Biochem Funct. 2026 Nov 15;44(11):e70343. doi: 10.1002/cbf.70343 (PMC13770262; doi:10.1002/cbf.70343)
Supplement: Supplementary file 4 [file CBF-44-e70343-s004.docx]

|  | **Species** | **Company** | **Catalogue Number** | **Dilution** |
| --- | --- | --- | --- | --- |
| **Primary Antibodies** |  |  |  |  |
| Ezrin Polyclonal antibody | Rabbit | Proteintech | 26056-1-AP | 1:100 |
| CoraLite®594-Phalloidin (red) |  | Proteintech | PF00003 | 1:100 |
| Anti-MiTF antibody | Mouse | Abcam | ab12039 | 1:500 |
| GAPDH Monoclonal Antibody | Rabbit | Cell Signaling Technology | 2118 | 1:1000 |
| MERTK Polyclonal antibody | Rabbit | Proteintech | 27900-1-AP | 1:1000 |
| Na+/K+ ATPase α-1 Antibody | Rabbit | Sigma-Aldrich | ZRB1574 | 1:5000 |
| SLC16A8 Polyclonal Antibody | Rabbit | Thermo Fisher Scientific | PA5-115908 | 1:1000 |
|  |  |  |  |  |
| **Secondary Antibodies** |  |  |  |  |
| Anti-Rabbit IgG (H+L), highly cross-adsorbed, CF™ 488A | Donkey | Sigma-Aldrich | SAB4600036 | 1:250 |
| Anti-Mouse IgG (H+L), highly cross-adsorbed, CF^™^ 647 | Donkey | Sigma-Aldrich | SAB4600176 | 1:500 |
| Anti-rabbit IgG, HRP-linked Antibody | Goat | Cell Signaling Technology | 7074 | 1:2000 |
| Anti-mouse IgG, HRP-linked Antibody | Horse | Cell Signaling Technology | 7076 | 1:2000 |

Table S1: List of Antibodies
